# Peptidoglycan potentiates the membrane disrupting effect of the carboxyamidated form of DMS-DA6, a Gram-positive selective antimicrobial peptide isolated from *Pachymedusa dacnicolor* skin

**DOI:** 10.1371/journal.pone.0205727

**Published:** 2018-10-16

**Authors:** Sébastien Cardon, Emmanuelle Sachon, Ludovic Carlier, Thierry Drujon, Astrid Walrant, Estefanía Alemán-Navarro, Verónica Martínez-Osorio, Dominique Guianvarc'h, Sandrine Sagan, Yannick Fleury, Rodrigue Marquant, Christophe Piesse, Yvonne Rosenstein, Constance Auvynet, Claire Lacombe

**Affiliations:** 1 Sorbonne Université, École normale supérieure, PSL University, CNRS, Laboratoire des Biomolécules, LBM, Paris, France; 2 Sorbonne Université, CNRS, Institut de Biologie Paris-Seine (IBPS), Plate-forme Spectrométrie de Masse et Protéomique, Paris, France; 3 Instituto de Biotecnologia, Universidad Nacional Autónoma de México, Cuernavaca, Morelos, México; 4 Université de Bretagne Occidentale, LUBEM EA, IUT Quimper, Quimper, France; 5 Sorbonne Université, CNRS, Institut de Biologie Paris-Seine (IBPS), Plate-forme de Synthèse Peptidique, Paris, France; 6 Faculté des Sciences et Technologie, Université Paris Est-Créteil Val de Marne, Créteil, France; VIT University, INDIA

## Abstract

The occurrence of nosocomial infections has been on the rise for the past twenty years. Notably, infections caused by the Gram-positive bacteria *Staphylococcus aureus* represent a major clinical problem, as an increase in antibiotic multi-resistant strains has accompanied this rise. There is thus a crucial need to find and characterize new antibiotics against Gram-positive bacteria, and against antibiotic-resistant strains in general. We identified a new dermaseptin, DMS-DA6, produced by the skin of the Mexican frog *Pachymedusa dacnicolor*, with specific antibacterial activity against Gram-positive bacteria. This peptide is particularly effective against two multiple drug-resistant strains *Enterococcus faecium* BM4147 and *Staphylococcus aureus* DAR5829, and has no hemolytic activity. DMS-DA6 is naturally produced with the C-terminal carboxyl group in either the free or amide forms. By using Gram-positive model membranes and different experimental approaches, we showed that both forms of the peptide adopt an α-helical fold and have the same ability to insert into, and to disorganize a membrane composed of anionic lipids. However, the bactericidal capacity of DMS-DA6-NH_2_ was consistently more potent than that of DMS-DA6-OH. Remarkably, rather than resulting from the interaction with the negatively charged lipids of the membrane, or from a more stable conformation towards proteolysis, the increased capacity to permeabilize the membrane of Gram-positive bacteria of the carboxyamidated form of DMS-DA6 was found to result from its enhanced ability to interact with peptidoglycan.

## Introduction

The World Health Organization (WHO) recently reported that infectious diseases account for nearly 13% of global deaths. By 2050, the continuous rise in antimicrobial resistance could lead to the death of 10 million people every year [1], notably due to an increase in hospital-related infections. Particularly, according to the WHO, Gram-positive bacteria are involved in major nosocomial infections, with 16% of these infections due to *Staphylococcus aureus*, one of the twelve families of bacteria considered a major threat for human health (http://www.who.int/mediacentre/news/releases/2017/bacteria-antibiotics-needed/en/). Furthermore, almost 38% of *S*. *aureus* strains are resistant to methicillin, and the number of *Staphylococcus* strains exhibiting resistance to vancomycin, one of the strongest available antibiotics, is increasing.

Despite the WHO considers antibiotic resistance a major issue of public health and prompts for the development of novel antibacterial molecules, major pharmaceutical companies have stopped antibiotic research due to the expanding rate of bacterial resistance and the slow approval of drugs. Over a period of twenty years (1995–2017), the number of antibacterial drugs approved by the United States Food and Drug Administration (FDA) has steadily decreased, and only twenty drugs have been approved for sale between 2008 and 2018 (https://www.centerwatch.com/drug-information/fda-approved-drugs/therapeutic-area/25/infections-and-infectious-diseases). Yet, the worldwide relentless emergence of antibiotic resistance continues to spur the search for novel anti-infectives to replace and/or supplement conventional antibiotics.

Since the discovery of the first antimicrobial peptide (AMP) in 1947, more than 2700 natural AMPs have been isolated from living prokaryotic and eukaryotic organisms (http://aps.unmc.edu/AP/main) [2]. Most peptides isolated to date are antimicrobial peptides with a broad spectrum against bacteria, fungi, and protozoa [3–5]. The antimicrobial activity of most AMPs is related to their capacity to bind to negatively charged bacterial surface molecules, mainly phospholipids, leading to permeabilization and disruption of the cell membrane [6, 7]. In this context, amidation of the C-terminus is generally thought to contribute to AMP activity because the peptide is less anionic than its carboxylate analogue, thus more attracted by the bacterial membrane [8–10].

Latin American frogs synthesize and secrete a wide range of biologically active small molecules and peptides with remarkable pharmacological properties. Among Hylidae and Ranidae frog skin AMPs, the most studied are those from the Dermaseptin family, a superfamily of 27- to 34-residue AMPs. In the present study, we isolated a member of the Dermaseptin family corresponding to DMS-DA6, a cationic peptide that had been previously identified [11] from the Mexican frog *Pachymedusa dacnicolor*. This peptide presents two naturally occurring versions, the acidic form (DMS-DA6-OH) and the corresponding amidated form (DMS-DA6-NH_2_) (Table 1 and S1 Fig). Herein, we show that both forms have specific antibacterial activity against Gram-positive bacteria, including antibiotic-resistant strains.

**Table 1:** Sequence, net charge at pH 7.4 and N- and C-terminus structures of DMS-DA6-OH and DMS-DA6-NH_2_.

Peptide	Sequence	Net charge at pH 7.4
DMS-DA6-OH	^+^H_3_N-GVWGIAKIAGKVLGNILPHVFSSNQS-COO-	+ 2
DMS-DA6-NH_2_	^+^H_3_N-GVWGIAKIAGKVLGNILPHVFSSNQS-CONH_2_	+ 3

Consistent with previous studies comparing carboxyamidated and carboxylated analogs [8, 9, 12–15], we also show that the bactericidal capacity of DMS-DA6-NH_2_ is higher than that of DMS-DA6-OH. However, in contrast to other studies, DMS-DA6-NH_2_ does not exhibit increased hemolytic activities, making this peptide a good candidate for new drug development. Understanding the mechanism of action of such peptide, and particularly the role of the C-terminal carboxyamidation that increases its antibacterial activity but not the hemolytic activity, could be a good starting point in the development of effective antibiotics. Previous studies have proposed that carboxyamidation enhances the interaction with the negatively charged bacterial membrane, inducing its permeabilization by increasing the net positive charge of the peptide and/or by stabilizing the membrane-bound structure [9, 16]. By exploring the structural properties and molecular interactions of the two analogs with Gram-positive bacteria membrane mimetics, we could not explain the more significant antibacterial activity of DMS-DA6-NH_2_ by (i) a more stabilized conformation, (ii) an increased positive net charge or (iii) different membrane-disrupting properties. Instead, the enhanced antibacterial activity of DMS-DA6-NH_2_ could be accounted for its capacity to interact with peptidoglycan, an essential component of the membrane of Gram-positive bacteria.

Altogether our data unravel that DMS-DA6 peptides act by strong perturbation of the bacterial membrane at different levels, highlighting a role for peptidoglycan as a potentiator of antimicrobial peptide function, and underscore the potential of DMS-DA6, particularly that of DMS-DA6-NH_2_, for developing new drugs against multi-resistant bacteria.

## Materials and methods

### Frogs, peptide isolation, purification and identification

Specimens of *Pachymedusa dacnicolor* were captured in the state of Morelos (Mexico) in the private land of YR, author of this study. *P*. *dacnicolor* is an endemic frog of the state of Morelos; it is not a frog species under threat of extinction according to the IUCN Red List of Threatened Species (http://www.iucnredlist.org/). Since frog specimens can be purchased legally in local pet stores in Mexico, no specific permissions were required for capturing and housing frogs. Mexican law does not require approval of the national Bioethics committee for experimental work on amphibians. Nonetheless, housing conditions and experimental procedures were approved by the Bioethics Committee of the Instituto de Biotecnología (Universidad Nacional Autónoma de México), and were undertaken by authorized investigators. Frogs were housed in a glass terrarium (70×40×40 cm) covered with a screen, close to a window, with normal daylight cycles, as previously described [17]. Substrate was soil, branches provided climbing space and natural local plants were added. Clean water was always available for the frogs to soak in. The terrarium was cleaned every other week and the plants and climbing structures renewed as needed. Frogs were fed with live crickets.

A full protocol describing isolation, purification, and sequencing of DMS-DA6 from the skin exudate of *Pachymedusa dacnicolor* is provided in the Supporting information section (S1 Protocol). Briefly, peptides isolation and purification from the frog exudate was performed by size exclusion chromatography on a Sephadex G-50 column followed by reverse phase high-performance liquid chromatography (RP-HPLC). Peptides identification was carried out by mass spectrometry analyses (S1 Protocol) of RP-HPLC collected fractions, directly or after acetylation [18]. Samples for MALDI-TOF (Matrix-assisted laser desorption/ionization-time of flight) analysis were prepared according to the “dried droplet” method [19].

### Solid-phase peptide synthesis

Peptides were synthesized by automated solid-phase peptide synthesis using Fmoc/tBu (9-Fluorenylmethoxycarbonyl/tertio-butyl) chemistry on an ABI 433A peptide synthesizer (Applied Biosystems) and purified by RP-HPLC (C18 Xbridge Waters, 5 μm 19 × 50 mm) using a solvent system composed of water containing 0.1% trifluoroacetic acid (TFA) as solvent A and acetonitrile containing 0.1% TFA as solvent B. The column was eluted at 10 mL.min^-1^ with a 35–60% linear gradient of solvent B for 10 min. Peptide purity was assayed by analytical HPLC (C8 Ace, 300Å, 5 μm, 250 × 4.6 mm, Ait France). Analytical chromatograms are shown in S2 Fig. Identities of the peptides were confirmed by MALDI-TOF mass spectrometry (MS Voyager Applied Biosystems). MS spectra of the purified peptides are shown in S3 Fig. Considering the presence of one tryptophan residue in the sequence, concentrations were determined by UV spectroscopy (Nanodrop, Fischer Scientific), assuming an extinction coefficient for Tryptophan ε_280_ = 5 600 M^-1^.cm^-1^.

Peptides were dissolved in water at a concentration of 1 mM and these stock solutions were further diluted in PBS (Phosphate buffered saline) or cell culture medium for biophysical and functional experiments.

### Antimicrobial assays

Gram-negative bacterial strains, *Escherichia coli* ATCC 8739, *Escherichia coli* ATCC 35218, *Escherichia coli* ML 35p, *Escherichia coli* K12, *Pseudomonas aeruginosa* ATCC 27853, and Gram-positive bacterial strains, *Enterococcus faecalis* ATCC29212, *Kocuria rhizophila* ATCC 9341, *Staphylococcus aureus* ATCC 6538, *Staphylococcus aureus* ATCC 29213, *Staphylococcus aureus* ST 1065, *Staphylococcus epidermidis* BM 3302, and *Bacillus subtilis* subsp. *subtilis* CIP 52.65, as well as two multiresistant strains (*Staphylococcus aureus* DAR 5829 (http://www.ncbi.nlm.nih.gov/bioproject/228435) and *Enterococcus faecium* BM 4147 [20] were cultured as described previously [21]. The minimal inhibitory concentration (MIC) of the peptides was determined by the microtiter broth dilution method. Overnight precultures were subcultured in Luria Bertani (LB) medium at 37°C with vigorous shaking until mid-log phase (~2h) and diluted to 10^7^ cfu/mL. Peptide solutions were prepared at different concentrations (from 2 to 500 μM) by successive 2-fold dilutions in water from 1 mM stock solutions. In each row of a 96-well nonbinding surface plate, 90 μL of bacteria were incubated with 10 μL of peptide solutions for 18 h at 37°C. The final peptide concentrations ranged from 0.2 to 100 μM. Cell growth was evaluated by visual inspection and absorbance at 600 nm to determine the lowest concentration of peptide that prevented cell growth. Wells were either opaque, indicating stationary phase growth, or transparent indicating no growth. The lowest concentration showing no visible growth was considered as the MIC. After measurement of the MIC, the bactericidal activity was determined by the addition of resazurin dye (30 μL, 0.01% w/v) to each well [22]. The plates were further incubated at 37°C for 18 h. Wells in which the dye turned pink indicated live bacteria, whereas wells in which the dye remained blue indicated dead bacteria. The minimal bactericidal concentration (MBC) is the lowest concentration with blue coloration (99.9% of bacteria were killed). Three to five independent assays were performed in triplicate. Positive controls correspond to medium plus 0.2% formaldehyde and negative controls to medium without peptide.

### Hemolytic activity

The hemolytic activity of both peptides was determined using fresh erythrocytes of mice or human healthy adult donors. The blood was centrifuged (800 × g for 7 min), and the erythrocytes were rinsed three times with PBS. Peptides at two concentrations (10 μM or 50 μM) were incubated with suspended erythrocytes (4% v/v in PBS) at 37°C for 60 min, and the cells were then removed by centrifugation at 800 × g for 5 min at 4°C. Hemolysis was assessed by measuring the supernatant absorbance at 550 nm. Erythrocytes lysed with 1% Triton X-100 were used as a standard for 100% hemolysis. The supernatant of an untreated erythrocyte suspension in PBS was used as blank.

### Stability in the supernatant of bacterial growth

Bacteria *S*. *aureus* ATCC 6538 were incubated as described for antimicrobial assays, with 6.5 μM DMS-DA6-NH_2_ or 12.5 μM DMS-DA6-OH (MIC/2) for 18 h at 37°C. After centrifugation (4,000 × g for 10 min), bacteria were discarded. The supernatant was filtered (syringe filter, pore size 0.2 μm) and incubated with 200 μM of either peptide at 37°C for 24 h. Intact peptides (time = 0) and incubated peptides were analyzed by reverse-phase HPLC. The experiment was performed on a Dionex ultimate 3000 system using a Higgins proto 200 C18 5 μm column. The mobile phase was a mixture of Solution A (0.1% TFA in water) and Solution B (0.1% TFA in acetonitrile), with an elution gradient of 0% to 80% Solution B over 10 min. The flow rate was 1 mL/min. Effluent absorbance was monitored at both 230 and 280 nm.

### Cytotoxicity experiments

A549 cells (ATCC CCL-185), a human lung carcinoma cell line, were cultivated at 37°C, humid atmosphere and 5% CO_2_, in Petri dishes with DMEM Advanced medium (Gibco) supplemented with 5% of fetal bovine serum (FBS), 2 mM of glutamine, 100 U of penicillin and 50 μg/mL of streptomycin (Gibco). When the cells reached 80% confluence, the medium was discarded and the cells were washed with PBS 1×, before being treated with Trypsin 1× for 2 minutes to detach them form the culture dish. Cells were then resuspended in DMEM medium, seeded in a 48-well culture plate (500 000 cells/well), and either form of DMS-DA6 solubilized in PBS 1× was added and incubated for 2 hours at 37°C, 5% CO_2_. DMSO 30% was used as a positive control to induce cell death, and DMEM medium as negative control. After incubation, cell viability was assessed by trypan blue exclusion.

### Bacterial membrane permeabilization assays

Membrane permeabilization was assessed by monitoring the hydrolysis of *ortho*-nitrophenyl β-D-galactopyranoside (ONPG) by the β-galactosidase enzyme, using the *S*. *aureus* ST1065 strain, specifically designed for membrane permeabilization assays, through detection of cytoplasmic β-galactosidase activity leakage [23]. Bacteria were grown in LB to an absorbance of 0.5 at 600 nm, pelleted by centrifugation, and the pellets were washed twice with PBS. Pellets were then resuspended in 10% LB in PBS to an absorbance of 0.5 at 600 nm. Aliquots (15 μL) of bacterial suspension were mixed with 2.5 mM ONPG and incubated with various concentrations of peptide in a 96-well plate. The hydrolysis of ONPG was monitored by measuring the absorbance at 420 nm of released *o*-nitrophenol with a Fluostar Galaxy microplate reader (BMG LabTech, France) [17].

### Bacterial lipid extraction

The bacteria (*E*. *coli* K12 strain or *S*. *epidermidis* BM3302 strain) were grown in batches of 8 L of LB at 37°C until OD_600_ = 0.6. Cells were collected by centrifugation (3,000 × g, 15 min, 4°C), and the pellets were re-suspended in 30 mL 20 mM Tris-HCl buffer, pH 8.0, centrifuged again and washed twice with the same buffer. Pellets were re-suspended in a mixture of methanol:chloroform (3 mL:1 mL/g of pellet). After agitating for 5 min, lipids were separated from the water-soluble material by diluting the extraction mixture with 1 mL/g of pellet of chloroform followed by 1 mL/g of pellet of water and agitated again for 4 min. The solution was centrifuged (4,000 × g, 12 min, 4°C), the organic phase was separated and the aqueous solution extracted twice with 15 mL chloroform. The organic phases were combined and dried under vacuum. The residue was solubilized in 3 mL toluene, centrifuged (20,000 × g, 1 min, 20°C) and the supernatant was dried under vacuum. The residue was dissolved in a minimal volume of chloroform and 10 volumes of acetone. The resulting phospholipid precipitate was separated through filtration on a sintered-glass filter and removed from the filter with small amounts of chloroform. The chloroform was evaporated and lipid vesicles were formed.

### Preparation of lipid vesicles

Lipid vesicles were formed using bacterial lipids extracted as described above or commercially available synthetic lipids. The lipids, 1-palmitoyl-2-oleoyl-*sn*-glycero-3-phosphocholine (POPC), 1-palmitoyl-2-oleoyl-*sn*-glycero-3-phospho-(1'-rac-glycerol) (POPG), 1,2-dimyristoyl-*sn*-glycero-3-phosphoglycerol (DMPG), 1.2-dioleyl-*sn*-glycero-3-phosphoglycerol (DOPG), and 1',3'-bis[1,2-dioleoyl-*sn*-glycero-3-phospho]-*sn*-glycerol (CL), were obtained from Avanti Polar Lipids, Inc (Alabaster, AL) and used without further purification. Desired mixtures of phospholipids were first dried in glass tubes under nitrogen and then maintained under a reduced pressure for at least 60 min. Lipid films were subsequently hydrated in PBS to yield a lipid concentration of 10 mg.mL^-1^ for tryptophan fluorescence experiments, 1 mg.mL^-1^ for DSC experiments, and in PB at 1 mM for CD experiments. The resulting dispersions of large multilamellar vesicles (MLVs) were subjected to 3 to 5 freeze-thaw cycles and then extruded 15 times through polycarbonate membranes (100 nm pore size, Whatman, Maidstone, UK) on an Avanti mini-extruder apparatus (Avanti Polar Lipids, Inc., Alabaster, AL) to generate large unilamellar vesicles (LUVs). Lipid vesicles were used either alone, or in combination with *S*. *aureus* peptidoglycan (PGN) (Sigma).

### Circular dichroism (CD) spectroscopy

The CD spectra of the peptides were recorded as previously described [24]. Measurements were carried out in 10 mM phosphate buffer at pH 7.4, 10 mM detergent (DPC or SDS), or in the presence of POPC, POPG and POPG-POPC (25:75) LUVs. Peptide concentration was 20 μM in all the samples, and measurements in the presence of lipids were performed with a peptide/lipid molar ratio of 1/50.

### Intrinsic tryptophan fluorescence

The fluorescence spectra of DMS-DA6 peptides were recorded from 295 to 475 nm with excitation at 280 nm, in a spectrofluorometer (Biologic Science Instruments ALX 250, MM 450, PMS 250, Software: Bio-Kine 32 V4.60, Grenoble, F). 150 μL of a 20 μM peptide solution were introduced in a 10 × 2 mm quartz cuvette (Hellman Analytics, Müllheim, GER) and increasing amounts of the lipid solution were added to obtain lipid concentrations of 10, 50, 200, 500, 1000, and 3000 μg.mL^-1^. After each addition, the tryptophan residue of the peptide was excited at 280 nm and the emission spectra were recorded from 300 to 480 nm. Spectra were normalized to take into account peptide dilution. We chose to calculate Δλmax−hence the difference between λ_max_ at any concentration of lipid and the λ_max_ of the peptide solution without added liposomes–and to plot the values against the concentration of lipid, as described by Hawrani and coworkers [25]. An apparent binding constant equivalent to the concentration of lipid at which the lipid-induced blue shift was half-maximal (K_L_) was thus determined.

### NMR spectroscopy and structure calculations

DMS-DA6 peptides were solubilized at a concentration of 1 mM in 550 μL H_2_O/D_2_O (90:10 v/v) in the presence of 80 mM SDS-*d*_*25*_ detergent (Eurisotop, Saint-Aubin, France). NMR experiments were recorded at 40°C on a 500 MHz Bruker Avance III spectrometer equipped with a TCI cryoprobe. ^1^H and ^13^C resonances were assigned from the analysis of 2D ^1^H-^1^H TOCSY, 2D ^1^H-^1^H NOESY (100 ms mixing time), and 2D ^1^H-^13^C HSQC spectra. NMR positioning experiments were recorded using the micellar samples of DMS-DA6 in the absence or presence of 1 mM gadodiamide. Paramagnetic relaxation enhancements were estimated from the *T*_*1*_ relaxation times of Hα protons as previously described [24].

Interproton distance restraints used for structure calculation were estimated from manually assigned NOEs collected on a 2D ^1^H-^1^H NOESY spectrum recorded at 800 MHz using a 60-ms mixing time. Three upper limit classes of 2.8, 3.8, and 5.0 Å were defined from the NOE cross-peak volumes. TALOS-N [26] was employed to derive ϕ and ψ dihedral angle restraints based on ^13^Cα, ^13^Cβ, ^1^H_N_, and ^1^Hα chemical shifts. A set of 100 structures was calculated by torsion angle dynamics in CNS [27] using standard parameters. The 20 lowest energy structures were retained and refined in an explicit layer of water, including an electrostatic energy term in the energy function. After the refinement process, the 12 structures exhibiting the lowest energies were selected to represent the NMR ensemble.

### Dextran leakage experiments

Rhodamine isothiocyanate (RITC)-labeled 10 kDa dextran-loaded liposomes were prepared by hydrating the dry lipid film with the buffer solution containing labeled dextran (5 mg.mL^-1^) as described by Rinaldi et al. [28] with some modifications. To achieve a better ratio of encapsulation, liposomes composed of cardiolipin (CL) and DOPG (40:60, w/w) to mimic Gram-positive membranes were submitted to a series of seven freeze-thaw cycles and LUVs (400 nm) were obtained as described above. Untrapped dextran was then removed by several cycles of centrifugation (21,500 × g for 40 min) and washing with PBS, until the residual fluorescence in the supernatant was negligible. Dextran release from loaded vesicles upon interaction with DMS-DA6 peptides was followed on a spectrometer (Biologic Science Instruments ALX 250, MM 450, PMS 250, Software: Bio-Kine 32 V4.60, Grenoble, F) by measuring the fluorescence of the supernatant obtained after centrifugation at 21500 × g for 40 min, after 5 min incubation with the peptide. The excitation wavelength was 550 nm, and emission spectra were recorded from 560 to 650 nm. 100% leakage was achieved by addition of Triton X-100 to a final concentration of 0.1%.

### Differential scanning calorimetry (DSC)

DSC experiments were performed on a NanoDSC microcalorimeter (TA instruments), using DMPG LUVs at a lipid concentration of 1 mg.mL^-1^. Peptides were added to the DMPG vesicles suspension at concentrations that allow a peptide to lipid ratio of 1/100, 1/50, and 1/25. A minimum of five heating and cooling scans (0–50°C, 1°C/min) was performed for each analysis. For experiments with PGN, the appropriate amount of a PGN suspension (40 μg.mL^-1^) was added to 1 mL DMPG-LUVs. The rest of the sequence is identical. DSC experiments were analyzed using the NanoAnalyze software provided by TA instruments. When needed, peaks were deconvoluted using the Modeling tool of NanoAnalyze.

## Results

### The carboxyamidated form of DMS-DA6 exhibits higher antibacterial activity against Gram-positive bacteria and is not hemolytic

In the search for novel biomolecules endowed with antimicrobial activity, fresh skin exudate from the Mexican frog *Pachymedusa dacnicolor* was fractionated (S4 Fig). Fraction I was further separated by RP-HPLC and RP-HPLC fractions were then tested for antibacterial activity. The fraction with a retention time of 55.7 min showed antimicrobial activity against the Gram-positive bacteria *S*. *aureus* ATCC 6538, but not against the Gram-negative bacteria *E*. *coli* ATCC 35218. The MALDI-TOF analysis of the RP-HPLC fraction revealed a peptide with a *m/z* ratio in the 2.0 to 3.5 kDa mass range, consistent with a Dermaseptin-like peptide (S5 Fig). Acetylation and *de novo* sequencing of this RP-HPLC fraction was performed to differentiate Lys from Gln residues before tandem MS fragmentation (MALDI-TOF/TOF) of the full-length parent ion. By combining the MS/MS information collected from the acetylated and non-acetylated parent peptides (S5 Fig), we obtained the sequence of DMS-DA6 (GVWGIAKIAGKVLGNILPHVFSSNQS), a 26-mer peptide previously identified through a proteomic study [11]. Our sequencing data unambiguously supported a free carboxyl terminus, while Meneses *et al*. [11] found the C-terminus of DMS-DA6 in the amidated form (DMS-DA6-NH_2_), suggesting that the two versions of DMS-DA6 may naturally co-exist in the frog exudate.

As reported in Table 2, both forms of DMS-DA6 were highly effective against a wide range of Gram-positive strains. Notably, they were active against the multidrug-resistant strains *Enterococcus faecium* BM4147 and *Staphycoloccus aureus* DAR 5829. DMS-DA6-NH_2_ was consistently more active than DMS-DA6-OH. On average, the MIC and MBC values obtained for DMS-DA6-OH were two to four-fold higher in comparison with DMS-DA6-NH_2_, suggesting that amidation of the *C*-terminus could be associated to a systematic increase in the antibacterial potency of DMS-DA6. Noteworthy, and contrary to a number of carboxyamidated peptides [29, 30], neither form of the peptide was hemolytic for human or murine erythrocytes at 10 μM or 50 μM (Table 2).

**Table 2:** Antimicrobial and hemolytic activities of DMS-DA6 peptides.

	Bacterial strain	Antimicrobial activity (MIC and MBC) (μM)			
		DMS-DA6-OH		DMS-DA6-NH_2_	
		MIC	MBC	MIC	MBC
Gram-negative	*E*. *coli* ML 35p	>100		>100	
	*E*. *coli* ATCC 35218	>100		>100	
	*E*. *coli* ATCC 8739	50	100	25	50
	*E*. *coli* K12	>100		>100	
	*Ps*. *aeruginosa* ATCC 27853	>100		>100	
Gram-positive	*S*. *epidermidis* BM3302	25	25	12.5	12.5
	*K*. *rhizophila* ATCC 9341	6.25	12.5	3.12	3.12
	*B*. *subtilis* CIP 52.65	12.5	12.5	6.25	6.25
	*S*. *aureus* ST 1065	25	25	12.5	12.5
	*S*. *aureus* ATCC 29213	6.25	25	3.12	6.25
	*S*. *aureus* ATCC 6538	12.5	12.5	6.25	6.25
	*S*. *aureus* DAR 5829*	50	50	25	50
	*E*. *faecalis* ATCC 29212	100	100	50	50
	*E*. *faecium* BM 4147*	50	100	25	25
		**Hemolysis (%)**			
		DMS-DA6-OH		DMS-DA6-NH_2_	
Human	10 μM	0.9 ± 1.5		1.2 ± 1.8	
	50 μM	7.4 ± 5.5		3.7 ± 3.3	
Mouse	10 μM	0 ± 2.8		0 ± 1.1	
	50 μM	5.4 ± 3.5		4.7 ± 5.6	

The MIC (minimal inhibitory concentration) and MBC (minimal bactericidal concentration) values are averaged over three independent experiments or more performed in triplicate. Strains with * are multi-resistant strains. Hemolytic activities were obtained by incubation of human or mouse erythrocytes in PBS with two concentrations of both peptides. The hemoglobin release was monitored by measuring the absorbance at 550 nm (Mean of 2 experiments).

### DMS-DA6-NH_2_ has a greater capacity than DMS-DA6-OH to permeabilize the *S*. *aureus* membrane

**Fig 1:**
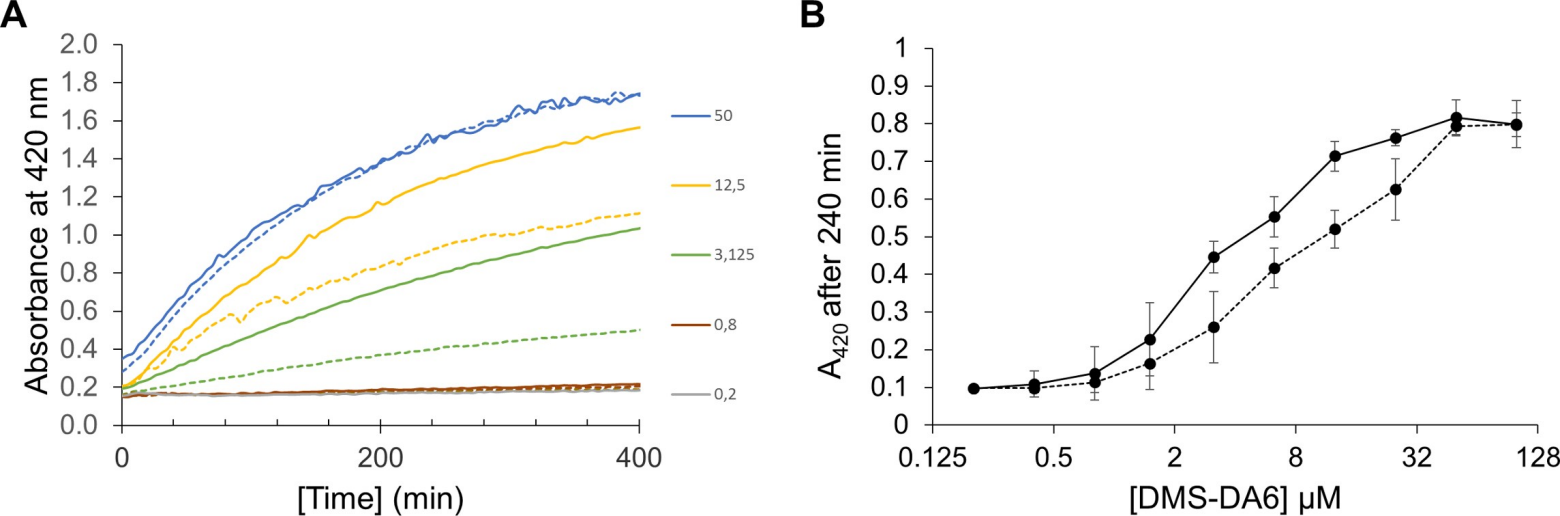
Membrane permeabilization of *S*. *aureus* ST 1065 as observed by ONPG hydrolysis. (A) Kinetics of bacterial membrane leakage after treatment with increasing concentrations (μM) of DMS-DA6-OH (dashed lines) and DMS-DA6-NH_2_ (solid lines). (B) The membrane leakage was followed by measuring ONPG hydrolysis at 420 nm after 240 min incubation with increasing concentrations of peptides. Data shown are representative of two independent experiments carried out in triplicate.

To outline the mechanism of action of these peptides, we assessed the ability of the two forms of DMS-DA6 to permeabilize the Gram-positive *S*. *aureus* membrane. As shown in Fig 1A, ONPG hydrolysis reached a plateau within 5 hours at the highest concentration (50 μM) of either form of the peptide. However, in consonance with the higher antibacterial activity of the carboxyamidated analog, ONPG hydrolysis was systematically higher for DMS-DA6-NH_2_ at concentrations within the 1.75–25 μM range, as illustrated in Fig 1B. The maximal absorption intensity observed after 240 minutes for DMS-DA6-NH_2_ was reached at a concentration of 12.5 μM, which corresponds to the minimal bactericidal concentration (MBC) determined for *S*. *aureus* ST 1065 (Table 2). The kinetics of *S*. *aureus* membrane leakage is slow in comparison with that of Gram-negative *E*. *coli* for which significant ONPG hydrolysis is usually detected within a few minutes after AMP addition. Such slow permeabilization kinetics of *S*. *aureus* membrane induced by DMS-DA6 was previously observed with other membrane-disrupting AMP belonging to the dermaseptin family [23]. Together, these results indicate that both DMS-DA6 peptides induced membrane leakage of Gram-positive bacteria, a result consistent with a cell lysis process mediated through peptide-membrane interactions.

**Fig 2:**
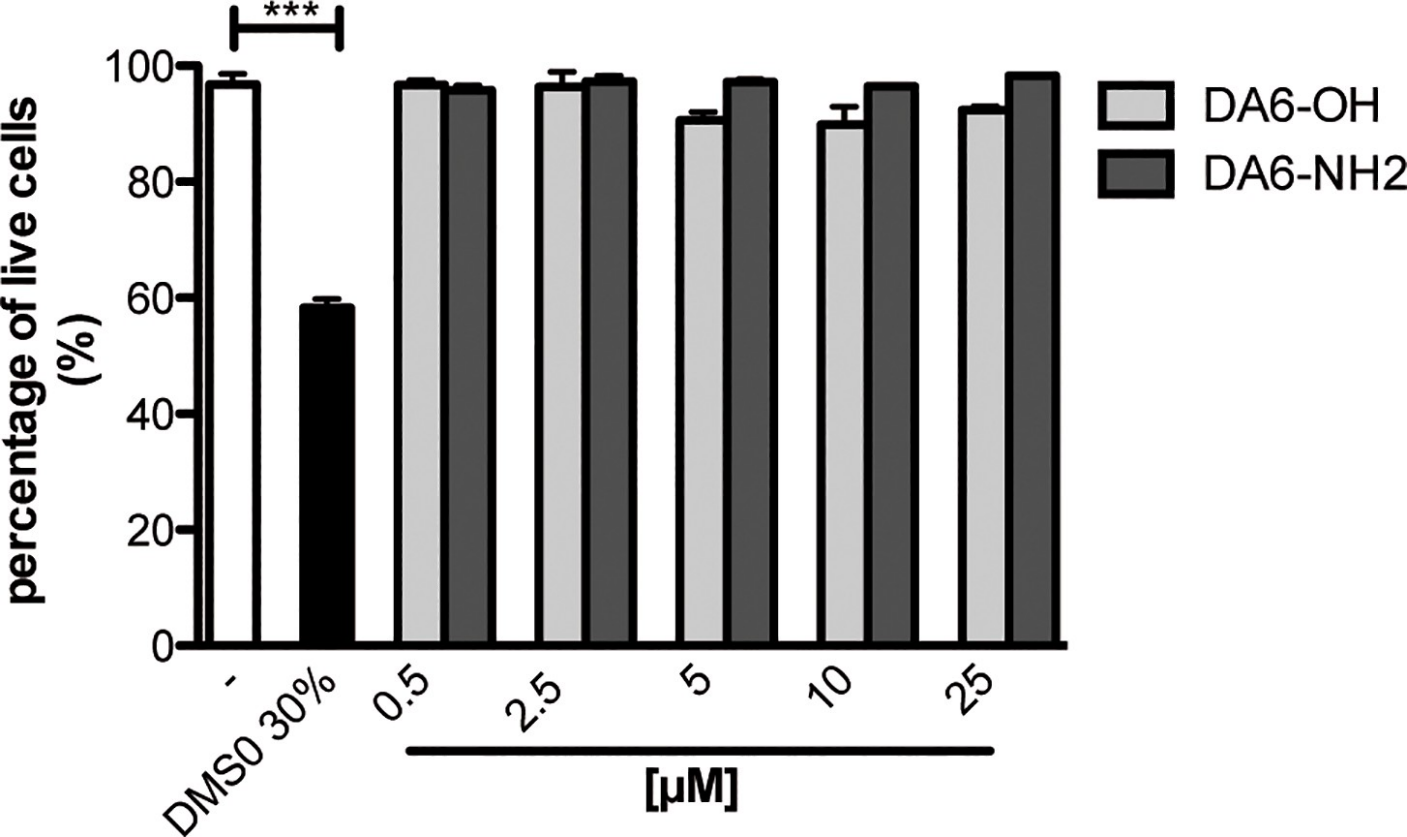
The DMS-DA6 peptides do not induce lysis of the negatively charged cancer cell line A549. Cells were incubated for 2 hours at 37°C, 5% CO_2_ in the presence of increasing concentrations of the peptides and the percentage of living cells was determined by trypan blue exclusion. Experiments were done in triplicate. Data represent the mean +/- SEM of 3 independent experiments run in triplicates.

Cancer cells tend to be more negatively charged than normal cells [31] and a variety of cationic antimicrobial peptides, including dermaseptins [32, 33], have been reported to exhibit antitumor activity. We thus investigated whether the peptides had distinct activities on cell viability, parallel to their antibacterial activities. The non-small-cell lung cancer cell lines A549 were incubated in the presence of DMS-DA6-OH or DMS-DA6-NH_2_. As shown in Fig 2, even after a 2-hour incubation with concentrations as high as 100 μM, neither DMS-DA6-OH (net charge +2) nor DMS-DA6-NH_2_ (net charge +3) resulted in the death of the negatively charged cancer cells.

Taken together, our results suggest that a negatively charged membrane is not a sufficient environment to induce membrane permeation, suggesting that other components than negatively charged lipids present on Gram-positive bacterial cell wall components may participate in DMS-DA6-NH_2_ higher activity.

### The increased membrane permeabilization capacity of DMS-DA6-NH_2_ does not result from a higher positive net charge, enhanced stability or structural differences in lipid and lipid-mimetic environments

**Fig 3:**
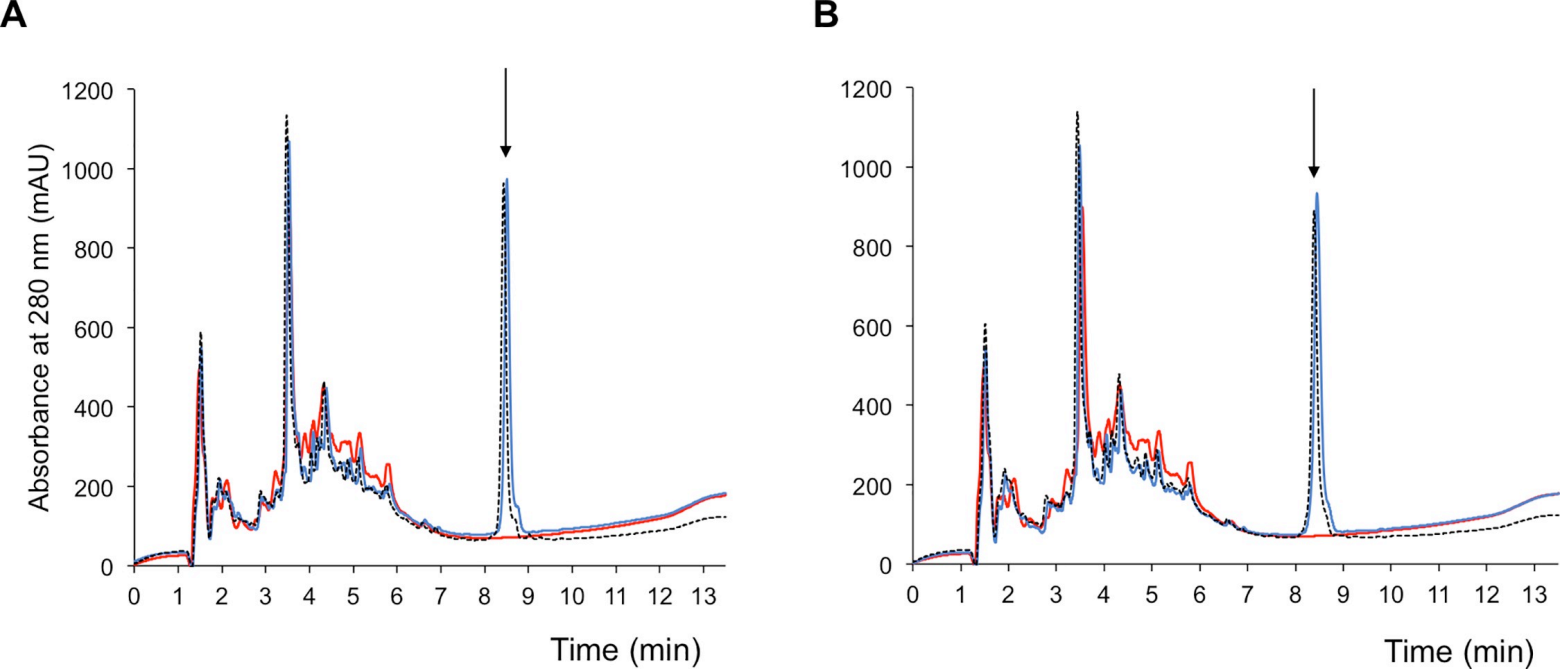
Stability of DA6 peptides in the bacterial supernatant. HPLC profiles of DMS-DA6-OH (A) and DMS-DA6-NH_2_ (B) at t = 0 (^**___**^) and t = 24h incubation (-—-). In red, HPLC profile of the incubation medium alone. The arrow points to the elution time of the intact peptide (8.5 min elution). Spectra shown are representative of three independent experiments.

To test the possibility that the C-terminal amidation may protect DMS-DA6-NH_2_ from proteolysis, thus increasing its antibacterial activity, as previously suggested for other peptides [16, 34, 35], we incubated the two versions of DMS-DA6 with bacterial culture medium for 24 hours and assessed their molecular integrity by HPLC. HPLC analysis revealed that more than 90% of either peptide remained intact after a 24-hour incubation period in the bacterial supernatant (Fig 3), thus precluding that the higher efficiency observed for the amidated version resulted from a higher proteolytic stability in culture medium.

**Fig 4:**
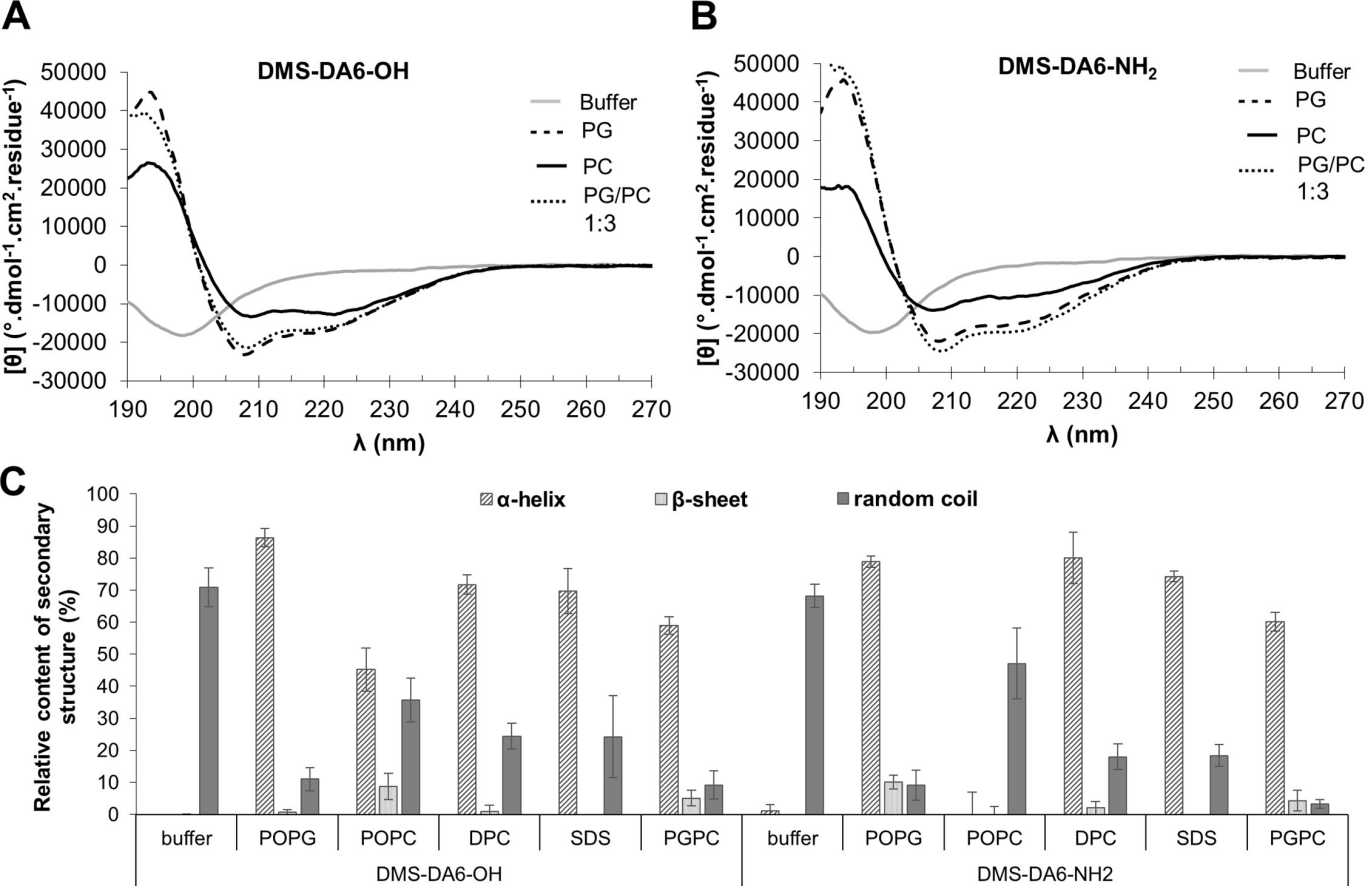
**Far-UV CD spectra of DMS-DA6-OH (A) and DMS-DA6-NH**_**2**_ **(B)** in phosphate buffer (―), POPG LUVs (-—-), POPC LUVs (―), and POPG/POPC (1:4) LUVs (^. . . . . . .^). (C) Conformations of DMS-DMS-DA6-OH and DMS-DA6-NH_2_ in the presence of various lipid vesicles as calculated from CD experiments. Data shown are representative of at least three different experiments.

Our next step was to determine the impact of carboxyamidation on the structural conformation of DMS-DA6 by CD and NMR. Antimicrobial peptides are generally unstructured in aqueous solution and tend to adopt a well-defined secondary structure in contact with lipid membranes. Accordingly, in phosphate buffer, the CD spectra of DMS-DA6-NH_2_ and DMS-DA6-OH show a single negative band near 200 nm, evidencing a random coil conformation of the peptides (Fig 4A and 4B). In contrast, in the presence of negatively charged POPG vesicles that mimic bacterial phospholipids, the two intense negative bands detected around 208 and 222 nm indicate that both peptides mostly adopted (about 80%) an α-helix conformation. In the presence of pure zwitterionic POPC vesicles, the contribution of α-helix conformation decreased to 40%. However, addition of a small proportion of negative charges, as in the mixture PC/PG (3:1) was sufficient to recover the high helical contents obtained with pure PG vesicles (Fig 4C), indicating that electrostatic interactions between the cationic residues of DMS-DA6 and the negatively charged lipids play a major role in stabilizing the helical structure. For all model membranes tested, the CD spectra of DMS-DA6-NH_2_ were very similar to those of DMS-DA6-OH, an observation that does not support a major influence of amidation on the overall secondary structure of DMS-DA6. Furthermore, the CD spectrum of DMS-DA6-OH obtained with DMPC/DMPG vesicles (S6 Fig) was highly similar to that recorded with POPC/POPG, suggesting that the nature of lipid acyl chains has little influence on DMS-DA6 secondary structure.

As CD spectra indicated similar helical contents in the presence of POPG vesicles and SDS micelles (Fig 4C and S6 Fig), the conformational properties of DMS-DA6-OH were investigated at the residue level by NMR spectroscopy in the presence of SDS. ^1^H and aliphatic ^13^C resonances were assigned from the analysis of ^1^H-^1^H TOCSY, ^1^H-^1^H NOESY, and ^1^H-^13^C HSQC correlation experiments. The deviation of Cα resonances (CSDs), defined as the differences between experimental chemical shifts and corresponding random coil values, were used as indicators of local conformational preferences. Most residues from Val-2 to Val-20 display positive CSD values above +1 ppm, characteristic of helical conformations (S7 Fig). The C-terminal region 22–26 appears rather unstructured, as inferred from the low CSD values between 0.1 and 0.5 ppm. Helical propensities were further confirmed by analysis of sequential and medium-range nuclear Overhauser effects (NOEs). In particular, the presence of a long helical region encompassing residues 3–21 is evident from the numerous dαN(*i*,*i+3*), dαβ(*i*,*i+3*), and dαN(*i*,*i+4*) medium-range NOE connectivities (S8 Fig).

**Fig 5:**
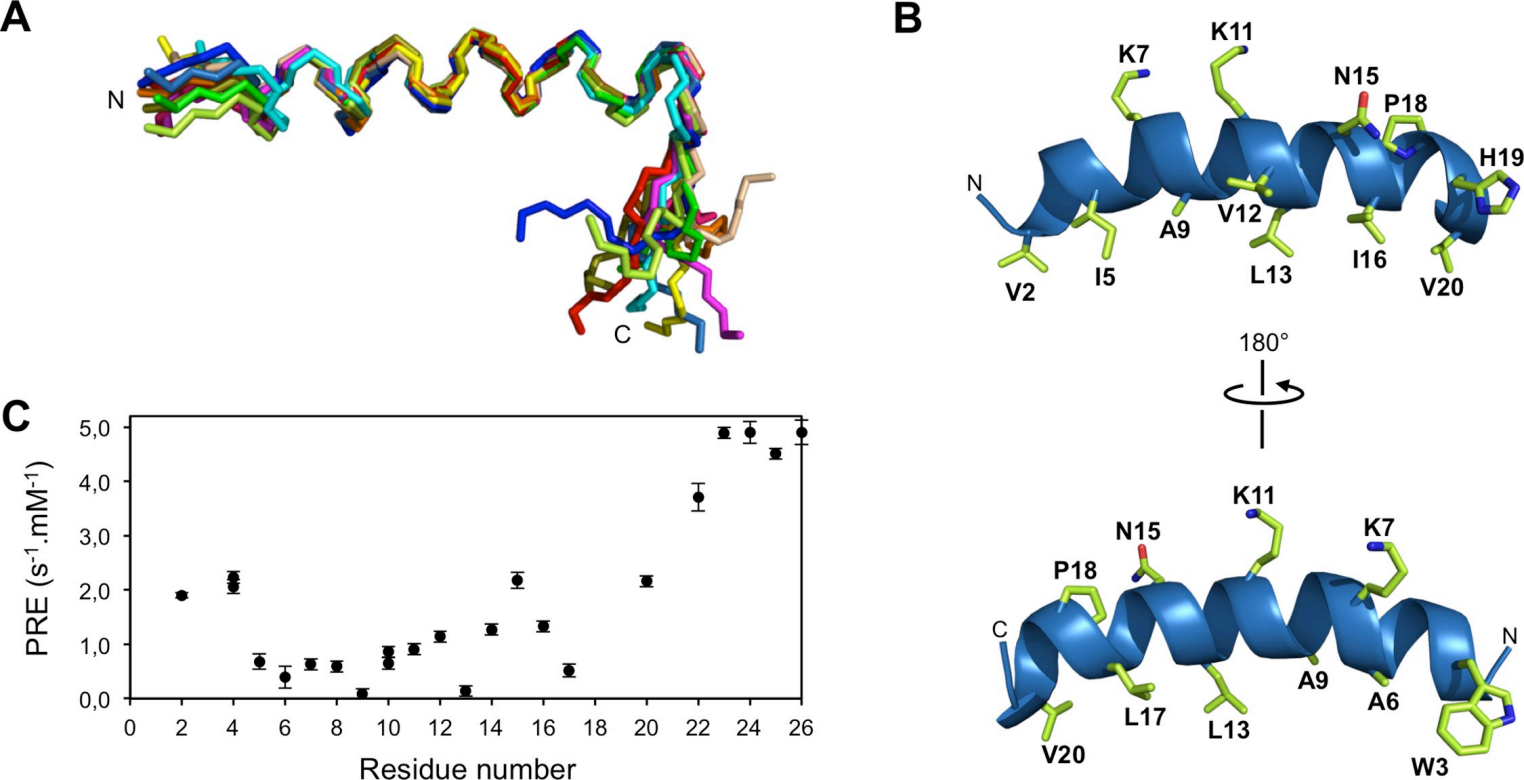
NMR structure of DMS-DA6 bound to SDS micelles. (A) Backbone superimposition of the 12 lowest-energy conformers over residues 3–21. (B) Ribbon representation of the central well-defined region of DMS-DA6 lowest-energy conformer. Side-chains of residues forming the hydrophobic and polar faces are represented by sticks. (C) Use of a paramagnetic reagent to probe the positioning of DMS-DA6 with respect to the micelle surface. Paramagnetic relaxation enhancements (PREs) induced by gadodiamide were measured as Hα relaxivities (differences in *R*_*1*_ longitudinal rates per mM concentration of gadodiamide).

The solution structure of DMS-DA6-OH was calculated in SDS micelles by restrained molecular dynamics, using distance and torsion angle constraints (S1 Table). The structure exhibits an uninterrupted helix in the central region 3–21 while terminal segments 1–2 and 22–26 are disordered (Fig 5A). The analysis of side chain distribution revealed a pronounced amphipathic character with a large hydrophobic face predominantly formed by Leu and Ile residues, along with Ala and Val amino acids (Fig 5B). The hydrophilic face is dominated by polar residues (particularly two cationic Lys) although it also contains apolar (Gly, Ile, and Pro) amino acids. The interfacial position of Trp-3 within such amphipathic topology is likely to be essential for the insertion of DMS-DA6 into bacterial membranes leading to cell disruption, as proposed for other antimicrobial peptides [36–38].

Surprisingly, although known as helix breakers, the Pro residue at position 18, together with the high content of Gly in the peptide sequence (4 over 26 residues) do not interrupt the helical conformation. Indeed, in water soluble globular proteins, Gly and Pro residues exhibit the lowest helical propensities and are often proposed to induce kinks or hinges in helical structures. The long helical region of DMS-DA6-OH does not show any pronounced bending but appears slightly curved towards the hydrophobic face (Fig 5B). A detailed inspection of the hydrogen bond networks indicates that backbone CO_i_-NH_i+4_ H-bonds, diagnostic of a α-helix, are observed throughout the helical structure, except near the Gly and Pro residues where such H-bonds are absent or show increased lengths. In particular, a CO_i_-NH_i+3_ H-bond involving the carbonyl group of Ile-16 is observed in the C-terminal region encompassing Pro-18. Interestingly, helical propensities are significantly lower at positions occupied by Gly residues and in the helical portion 15–20, as supported by decreasing CSD values (S7 Fig). Overall, these observations suggest that the α-helical distortions of DMS-DA6-OH likely result from the presence of helix-destabilizing residues in the peptide sequence.

The position of DMS-DA6 with respect to the micelle surface was examined with gadodiamide, a water-soluble paramagnetic probe that partitions exclusively outside the micelle. The addition of gadodiamide led to differential paramagnetic relaxation enhancements (PREs) of Hα protons (Fig 5C). Residues in the central portion of the helical region displayed the lowest PREs indicating that they are buried inside the micelle. Periodicity in the PRE values was apparent in the region 5–17 supporting a parallel positioning of this central portion with respect to the micelle surface. Apolar residues Ala-6, Ala-9, Leu-13, and Leu-17, which lie on the hydrophobic face, were the least affected by gadodiamide, demonstrating that these residues orient their side chains towards the micelle interior. Although located on the hydrophobic face, residue Val-20 is substantially affected by the paramagnetic probe and is therefore positioned close to the water-micelle interface. A higher sensitivity to gadodiamide may also result from an increased flexibility at the helix C-terminus induced by Pro-18.

**Fig 6:**
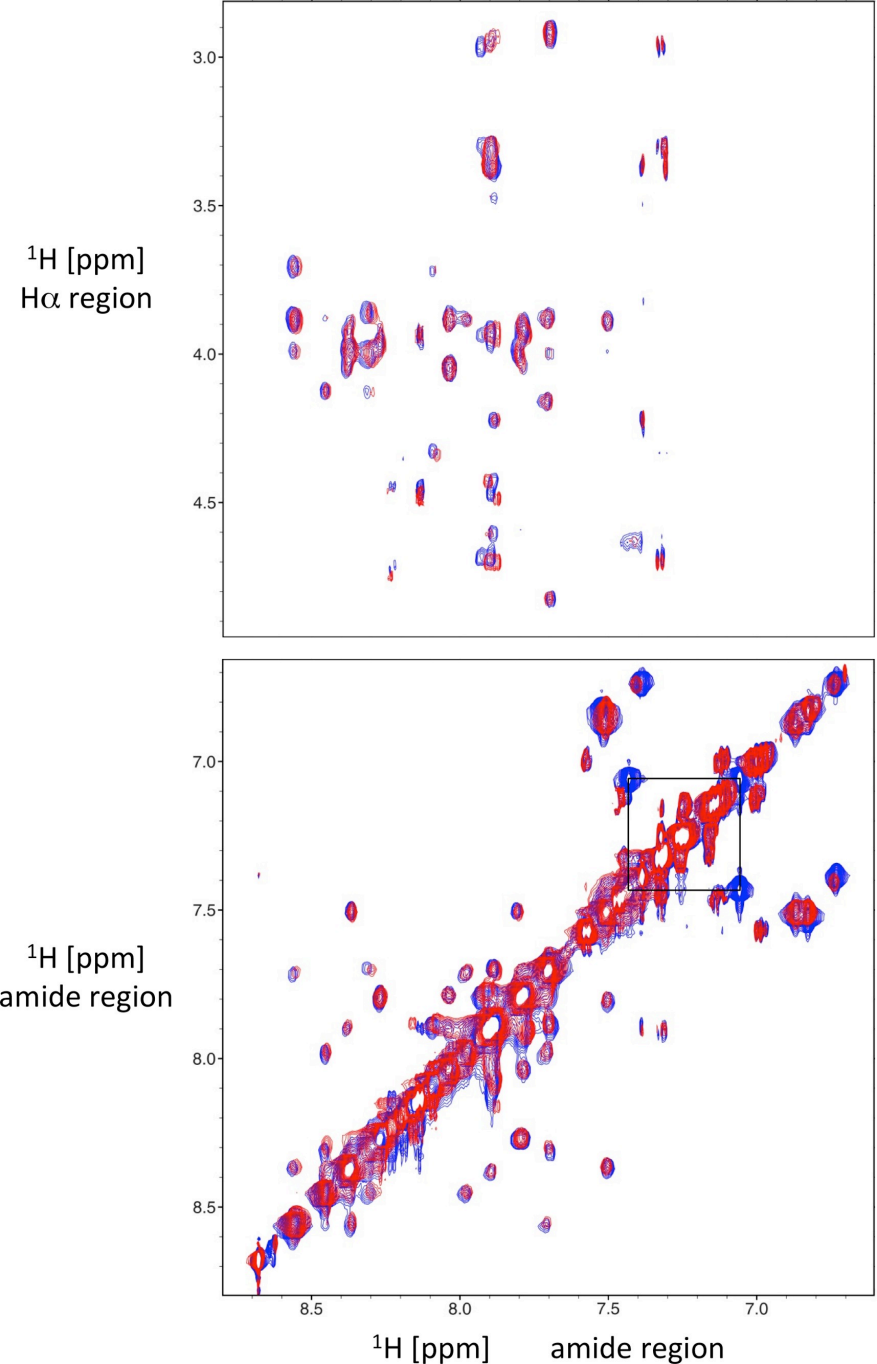
Overlay of 2D ^1^H-^1^H NOESY spectra recorded at 500 MHz on DMS-DA6-OH (red) and DMS-DA6-NH_2_ (blue) in the presence of SDS micelles. Additional cross-peaks corresponding to the correlations between the two protons of the C-terminus amide group in DMS-DA6-NH_2_ are indicated by a black square.

The NMR structure of DMS-DA6-OH in SDS micelles is consistent with the high helical content of both peptide forms in POPG and POPC/POPG (4:1) vesicles, assessed by CD spectroscopy. The 2D NOESY spectrum of DMS-DA6-NH_2_ in SDS micelles overlaid almost entirely with that of DMS-DA6-OH (Fig 6), further confirming that the C-terminal amidation does not affect the structure of the peptide.

Altogether, the CD and NMR studies uncovered the α-helix conformation of membrane-bound DMS-DA6 but did not reveal an influence of the C-terminal amidation on the structure of the peptide. As the central region 5–17 of both peptides can insert deeply into the hydrophobic core of micelles, we still lacked an explanation for the superior ability of DMS-DA6-NH_2_ to disrupt bacterial membrane, compelling us to investigate this further.

### DMS-DA6-NH_2_ enhanced activity is not an effect of stronger interactions with negatively charged membranes

**Fig 7:**
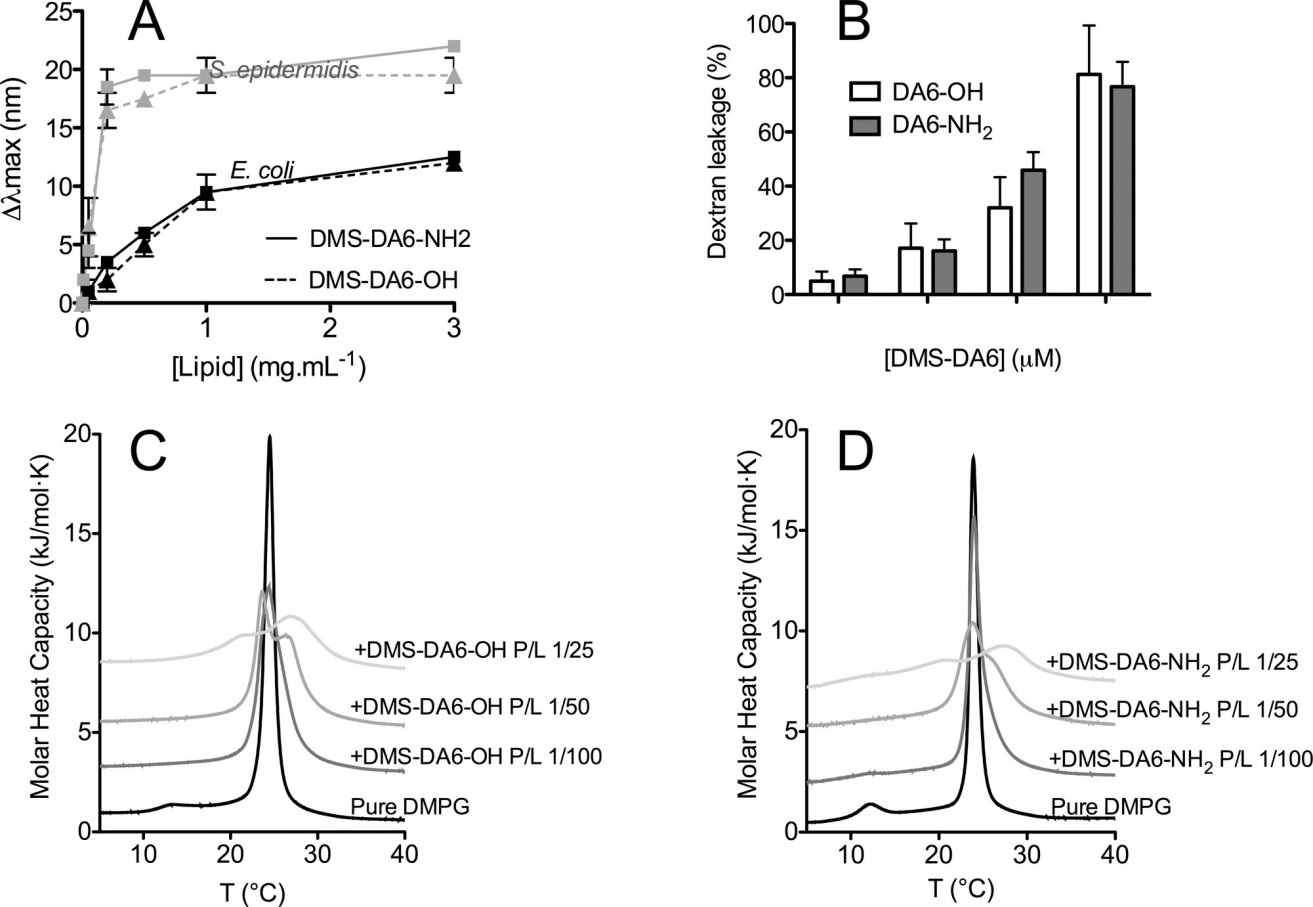
Perturbation of membranes or lipids induced by interaction with DMS-DA6 peptides. (A) Lipid-induced change in DMS-DA6 tryptophan fluorescence. Blue shift (Δλ_max_) for tryptophan in the wavelength of maximal emission in the presence of LUVs of liposomes produced from *E*. *coli* phospholipids (black) or from *S*. *epidermidis* phospholipids (grey) in the presence of DMS-DA6-NH_2_ (^__^) or DMS-DA6-OH (-—-). (B) Dextran leakage from DOPG/CL LUVs after treatment with increasing concentrations of DMS-DA6. (C) and (D) DSC themograms obtained after addition of DMS-DA6-OH (C) or DMS-DA6-NH_2_ (D) to DMPG LUVs. Data shown are representative of 3 different experiments.

The fluorescence emitted by aromatic tryptophan residues of peptides and proteins varies depending on the polarity of the environment, and is thus informative of the position of tryptophan-containing peptides in lipid membranes [39]. To evaluate if carboxyamidation influenced the capacity of DMS-DA6 to interact with and insert itself into membranes with different composition of phospholipids, we explored the tryptophan fluorescence spectra of DMS-DA6-OH and DMS-DA6-NH_2_ in the presence of phospholipids extracted from Gram-positive (*S*. *epidermidis*) or Gram-negative (*E*. *coli*) bacteria. As expected, when in aqueous buffer, the tryptophan fluorescence spectra of DMS-DA6-NH_2_ and DMS-DA6-OH had a maximum emission at 355 nm. The addition of increasing concentration of LUVs prepared with *E*. *coli* or *S*. *epidermidis* phospholipids resulted in a substantial blue-shift in the emission maxima of either peptide, suggestive of the incorporation of the tryptophan aromatic ring into the hydrophobic environment of the negatively charged lipids (Fig 7A). DMS-DA6-NH_2_ and DMS-DA6-OH interacted with and inserted into *S*. *epidermidis* phospholipid membranes, with a blue-shift of around 20 nm and an apparent binding constant K_L_ around 0.02 mg.mL^-1^. In contrast, a weaker interaction with *E*. *coli* phospholipid membranes was observed, with a blue-shift of around 10 nm and weaker K_L_ values of 0.72 and 0.95 mg.mL^-1^ for DMS-DA6-OH and DMS-DA6-NH_2_, respectively (Fig 7A, Table 3). These data suggest that the phospholipid composition of the membrane is crucial for the interaction and insertion of DMS-DA6 into the membrane and that both forms of DMS-DA6 have a higher affinity for Gram-positive bacteria phospholipids, consistent with the antibacterial assays results. However, the greater membrane permeabilization capacity of the carboxyamidated form of DMS-DA6 was not dependent on the nature of the lipids present in the membrane.

**Table 3:** Apparent binding constants K_L_ for the interaction of DMS-DA6 peptides with phospholipids extracted from *S*. *epidermis* and *E*. *coli* bacteria determined by tryptophan fluorescence.

	Bacterial strain lipids		
K_L_ (mg.mL^-1^)	*E*. *coli*		*S*. *epidermidis*
**DMS-DA6-OH**	0.95 ± 0.10		0.02 ± 0.005
**DMS-DA6-NH**_**2**_	0.72 ± 0.04		0.02 ± 0.002

The apparent binding constant K_L_ is defined as the lipid concentration that induces 50% of maximal blue-shift.

We next investigated membrane defects induced by the DMS-DA6 peptides using dextran leakage assays. Peptide-induced liposome leakage was monitored by measuring the fluorescence of rhodamine-labelled dextran released into the supernatant after centrifugation. Dextran leakage from the liposomes can be directly related to the lytic effect of the peptide. Release of 10 kDa dextran corresponds to pores or membrane defects larger than 2.3 nm, according to Stokes radius data provided by the manufacturer. Fig 7B shows that dextran release, calculated as a percentage of the release induced by 0.1% Triton X-100, was peptide concentration dependent, with a maximum obtained for a peptide/lipid molar ratio 1/1000. These results indicate that the pores created by DMS-DA6 peptides have an estimated radius of at least 2.3 nm, and are likely responsible for their antimicrobial activities.

We further evaluated the interaction of the two forms of DMS-DA6 with lipids by DSC. As complex lipid mixtures are not well adapted to DSC analysis, we use pure DMPG to mimic Gram-positive bacterial membranes. Thermograms illustrating the effect of incorporating increasing quantities of DMS-DA6-NH_2_ and DMS-DA6-OH are presented in Fig 7C and 7D, and the corresponding thermodynamic parameters are provided in Table 4. Pure DMPG vesicles display two distinct endothermic transitions: a less energetic event around 13°C corresponding to the transition between the Lβ’ gel phase to the Pβ’ ripple phase, the so-called pre-transition, and a more energetic event around 24°C corresponding to the transition between the Pβ’ripple phase to the Lα fluid phase, the so-called main transition. Addition of either version of DMS-DA6 completely abolished the pre-transition, even at the lower peptide to lipid ratio (P/L) of 1/100, indicative of a strong interaction between the peptide and the lipid head-groups. At this peptide concentration, the main transition peak was minimally affected, except for some tailing towards higher temperatures, possibly explaining the slight increase in the transition enthalpy. Increasing the peptide concentration to P/L = 1/50 resulted in a clear splitting of the transition peak, suggesting that the peptides promoted the lateral segregation on the bilayer, creating peptide-rich and peptide-poor regions, as has been reported for basic arginine-rich peptides [40]. Furthermore, this was accompanied by a general decrease of the transition enthalpy, pointing to an interaction between the peptides and the fatty acid chains of the lipids. The high-temperature peak, with a higher transition enthalpy closer to that of pure lipid, probably corresponds to a peptide-poor region. The low-temperature peak has a much lower transition enthalpy that would correspond to the emerging peptide-rich region. As peptide concentration increased, the two peaks were more separated, and the enthalpy transition further decreased. This data indicates that both versions of DMS-DA6 equally disorganize a DMPG bilayer, consistent with the bacterial and model membrane leakage experiments, but still fails to provide an explanation for the substantially higher antibacterial activity of the carboxyamidated form of the peptide.

**Table 4:** Thermodynamic parameters obtained by DSC corresponding to DMS-DA6 peptides interacting with DMPG LUVs at different peptide to lipid ratios.

	T_m1_ (°C)	ΔH_m1_ (kJ/mol)	T_m2_ (°C)	ΔH_m2_ (kJ/mol)
Pure DMPG	24.1 ± 0.1	24.7 ± 1.3	-	-
+ DMS-DA6-OH, 1/100	24.0 ± 0.2	28.2 ± 1.5		
+ DMS-DA6-OH, 1/50	25.9 ± 0.3	18.5 ± 2.5	23.3 ± 0.3	5.4 ± 1.2
+ DMS-DA6-OH, 1/25	27.8 ± 0.6	11.5 ± 2.3	14.7 ± 3.3	2.6 ± 1.8
+ DMS-DA6-NH_2_, 1/100	23.9 ± 0.2	29.6 ± 3.7	-	-
+ DMS-DA6-NH_2_, 1/50	25.8 ± 0.1	15.7 ± 1.6	22.9 ± 0.6	5.6 ± 2.2
+ DMS-DA6-NH_2_, 1/25	28.0 ± 0.4	9.6 ± 1.6	16.1 ± 4.0	2.0 ± 0.3

### DMS-DA6-NH_2_ interacts with peptidoglycan, a major component of Gram-positive bacteria cell wall, amplifying membrane permeabilization

Apart from phospholipids, peptidoglycan (PGN) is a major neutral molecule present on the membrane of Gram-positive bacteria. Malanovic and Lohner [7] recently suggested that peptidoglycan could function as a cell wall sponge, attracting peptides to the surface of the bacterial lipid membrane, thus potentiating the action of antimicrobial peptides by increasing their local concentration at the level of the bacterial membrane. We investigated whether peptidoglycan could contribute to the greater activity of DMS-DA6-NH_2_ compared to DMS-DA6-OH by using lipid vesicles containing peptidoglycan.

**Fig 8:**
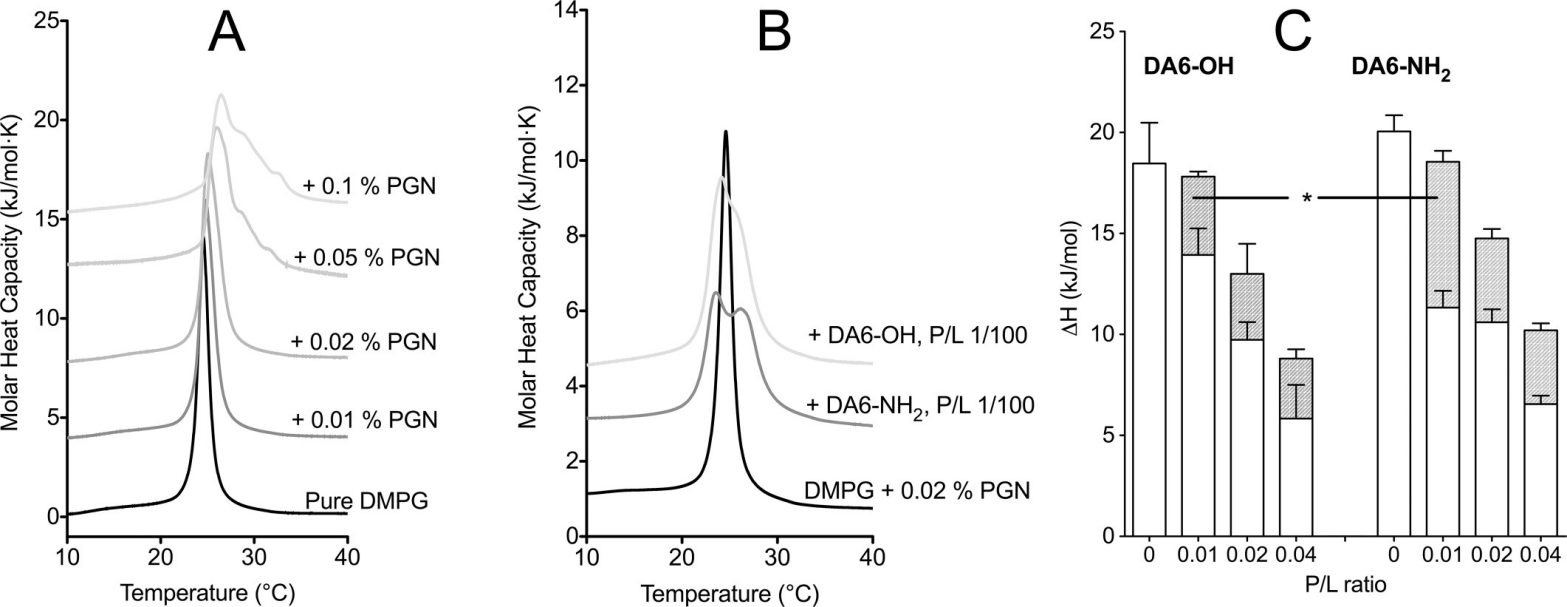
PGN enhances the membrane permeabilization capacity of DMS-DA6. **(**A) Thermograms of DMPG LUVs according to PGN concentration. (B) Thermograms showing the effect of the addition of DMS-DA6 peptides to DMPG/PGN LUVs at P/L = 1/100. (C) Transition enthalpy obtained by DSC for the “peptide-poor” (white) and “peptide-rich” (dashed) contributions to the main transition peak for the interaction of DMS-DA6 peptides with DMPG/PGN LUVs. The bars represent mean ± SEM, n ≥ 3. Significance was tested using a t-test, *, p = 0.0042.

We first defined the optimal amount of peptidoglycan to be used to avoid a complete perturbation of the DSC thermograms. Consistent with a previous report [41], even though the transition enthalpy remained unchanged, as peptidoglycan concentration augmented, the main transition peak shifted to higher temperatures and became broader, with the appearance of shoulders at higher temperatures (Fig 8A). Experiments were thus carried out with DMPG-LUVs with 0.02% (w/w) peptidoglycan since at this concentration the transition peak is still unique, though slightly broader than with pure DMPG. DMS-DA6 peptides were then gradually added to the DMPG-peptidoglycan LUVs. The corresponding data are reported in Table 5.

**Table 5:** Thermodynamic parameters obtained by DSC corresponding to DA6 peptides interacting with DMPG LUVs + PGN at different peptide to lipid ratios.

	T_m1_ (°C)	ΔH_m1_ (kJ/mol)	T_m2_ (°C)	ΔH_m2_ (kJ/mol)
DMPG + PGN	24.61 ± 0.06	19.4 ± 0.9	-	-
+ DMS-DA6-OH, 1/100	25.55 ± 0.05	13.9 ± 1.3	23.3 ± 0.2	3.9 ± 0.2
+ DMS-DA6-OH, 1/50	28.7 ± 1.8	9.7 ± 0.9	18.3 ± 3.9	3.3 ± 1.5
+ DMS-DA6-OH, 1/25	29.9 ± 3.1	5.8 ± 1.7	14.2 ± 2.9	3.0 ± 0.5
+ DMS-DA6-NH_2_, 1/100	26.8 ± 0.2	11.3 ± 0.9	23.6 ± 0.2	7.2 ± 0.5
+ DMS-DA6-NH_2_, 1/50	28.3 ± 0.3	10.6 ± 0.6	21.4 ± 0.5	4.2 ± 0.5
+ DMS-DA6-NH_2_, 1/25	27.9 ± 0.2	6.6 ± 0.4	11.2 ± 0.2	3.7 ± 0.4

Similar to experiments performed in the absence of peptidoglycan, the addition of either form of DMS-DA6 to the DMPG/PGN LUVs led to the splitting of the main transition peak. However, different to what we observed without peptidoglycan, this happened even at the lowest P/L ratio (Fig 8B). Furthermore, at a P/L ratio of 1/100, the lower temperature “peptide-rich” peak was considerably bigger with DMS-DA6-NH_2_ than with DMS-DA6-OH (Fig 8C), although this difference was no longer significant at higher peptide concentrations. Collectively, this data suggests that in the presence of peptidoglycan, DMS-DA6-NH_2_ is more efficiently disrupting the membrane than DMS-DA6-OH, providing a plausible explanation for the better performance of DMS-DA6-NH_2_ over DMS-DA6-OH on Gram-positive bacteria.

## Discussion

In the search for novel antimicrobial agents, we isolated a member of the Dermaseptin family corresponding to DMS-DA6 (GVWGIAKIAGKVLGNILPHVFSSNQS) from the Mexican frog *Pachymedusa dacnicolor*. Different to most Dermaseptins that share a Trp residue at position 3 and a conserved motif (AAXKAALXA) in the central region of the molecule [42], DMS-DA6 lacks the alanine-rich central motif. Furthermore, in contrast to a previous publication reporting that the C-terminus of DMS-DA6 was amidated (DMS-DA6-NH_2_) [11], our sequencing data unambiguously supported a free carboxyl terminus, thus suggesting that these two versions of DMS-DA6 may naturally co-exist in the frog exudate.

Although Dermaseptins typically display a broad-spectrum activity against both Gram-positive and Gram-negative strains [43], we found that both forms of the DMS-DA6 peptide were highly specific against Gram-positive bacteria. Notably, they were active against the multidrug-resistant strains *Enterococcus faecium* BM4147 and *Staphylococcus aureus* DAR 582. Interestingly, AMPs with the closest sequences to DMS-DA6 (S2 Table), *i*.*e*., the three plasticins PD36K, PD36, B1 [44], and CPF-St5 from *Sirulina tropicalis* [45] exhibit broad-spectrum antibacterial activities, featuring DMS-DA6 as a promising peptide for the design of new antibiotics, especially against multi-resistant strains.

Amidated peptides are frequently more resistant to enzyme degradation, and also more active than their non-amidated counterparts [8, 9, 12–14, 16]. The observation that the amidated form of the DMS-DA6 was invariably 2- to 4-fold more potent than the C-terminal acidic form compelled us to further analyze the functional and structural properties of both forms of DMS-DA6.

Our structural studies showed that a small proportion of negatively charged lipids (POPG) sufficed to trigger the transition of both DMS-DA6 peptides from random coil to α-helix, in agreement with the concept that ionic interactions promote folding, as reported for magainins [16, 35, 46]. Interestingly, unlike other antimicrobial peptides with low cytotoxicity on mammalian cells [47], the proline and glycine residues in DMS-DA6 do not disrupt the helical fold of the membrane-bound structure. However, there was no difference in helix formation or stability in bacterial culture medium between the two variants of DMS-DA6.

As the bacterial membrane leakage experiments pointed towards the membrane bilayer as the primary target for the DMS-DA6 peptides, we investigated whether the two forms of the peptide interacted differently with model membranes, using lipids extracted from microorganisms alongside with synthetic lipid models. The results from the dextran leakage experiments were in line with the bacterial membrane leakage experiments, suggesting that both forms of the DMS-DA6 peptide produce pores in the membrane, large enough to let 10 kDa dextran or β-galactosidase leak through, in a concentration-dependent manner. Furthermore, data of the tryptophan fluorescence done with LUVs made with Gram-positive membrane lipids indicate that the tryptophan residue transitions from a polar environment to a non-polar-one, suggesting increasing contacts of both forms of the DMS-DA6 peptide with the lipid chains. The smaller blue-shift observed in the presence of *E*. *coli* lipids LUVs (around 10 nm) is consistent with a partial insertion of DMS-DA6 into this vesicle type, further accounting for the selectivity towards Gram-positive bacteria. Overall, DSC experiments confirmed that both DMS-DA6 peptides could insert into and disorganize a membrane composed of anionic lipids. However, the greater interaction and insertion of the carboxyamidated form of DMS-DA6 was not dependent on the nature of the lipids or on the negative charge of the membrane, as supported by the low toxicity of either form of DMS-DA6 on negatively charged mammalian cancer cells.

As proposed by Brannan et al.[48], many AMPs must bind to non-lipid components of the cell to account for the discrepancy between the amounts of peptide needed to observe disruption in model membranes versus bacteria. Indeed, the relative leakage induced in model lipid membranes by different AMPs does not systematically correlate with their relative capacity to prevent bacterial growth [49], suggesting that AMPs may have alternate modes of action. Following the recent observations of Malanovic and coworkers proposing that peptidoglycan acts as a mesh that could enhance the activity of the AMP OP-145 [7, 41], we assessed the possibility that peptidoglycan may also contribute to the ability of the DMS-DA6 peptides to disrupt Gram-positive bacterial membranes. In the presence of peptidoglycan, the membrane perturbation induced by either form of DMS-DA6 was considerably more severe than in the absence of peptidoglycan, even at the lower 1/100 P/L ratio. Furthermore, at this peptide/lipid ratio, DMS-DA6-NH_2_ was significantly more active than DMS-DA6-OH to induce lateral segregation. As the P/L ratio increased, the difference between the two forms of the peptide tended to diminish, with DMS-DA6-NH_2_ being consistently more effective than DMS-DA6-OH at intermediate concentrations. Moreover, experiments with *Enterococcus faecium* BM4147, a peptidoglycan modified strain [20], resulted in greater antimicrobial activities in comparison with a wild-type *E*. *faecalis* strain (Table 2). This is particularly interesting as the PGN modification in *E*. *faecium* BM4147 leads to vancomycin resistance. Consistent with a recent report [50] showing that melittin and cecropin A interact with peptidoglycan, our data strongly support a role for peptidoglycan as a potentiator of DMS-DA6-NH_2_ activity, likely by trapping the peptide close to the bacterial membrane, increasing its local concentration, thus amplifying its action. Since the C-terminal part of both DMS-DA6 peptides is highly flexible, the carboxylate moiety could act as a repulsive group and curb binding of DMS-DA6-OH to PGN, a situation not occurring for DMS-DA6-NH_2_. How DMS-DA6 peptides could interact with PGN at a molecular level remains to be investigated.

In conclusion, DMS-DA6 is a promising new AMP, highly specific against Gram-positive bacteria and devoid of hemolytic activity. We demonstrated that in addition to its interaction with the bacterial membrane lipids, DMS-DA6 also interacts with peptidoglycan, a major component of the membrane of Gram-positive bacteria, possibly explaining the specificity of DMS-DA6 for Gram-positive bacteria. Notably, the carboxyamidated form of the peptide exhibited enhanced permeabilization capability resulting from a better capacity to interact with peptidoglycan, potentiating its anti-bacterial efficiency. All these particular features make this non-conventional Dermaseptin a good candidate for developing new therapeutic peptides against multiresistant bacteria.

## Supporting information

S1 Fig Chemical structure of DMS-DA6 peptides. (TIF)

S2 Fig Analytical HPLC chromatograms of synthetized DMS-DA6 peptides. (TIF)

S3 Fig MALDI-TOF reflector positive ions analysis of synthetized DMS-DA6 peptides. (TIF)

S4 Fig Isolation and purification of natural DMS-DA6 from the skin exudate of *Pachymedusa dacnicolor*. Fractionation profile of the skin exudate on a Sephadex G-50 column followed by reverse-phase HPLC separation using a semi-preparative column.
(TIF)

S5 Fig Sequencing of natural DMS-DA6 secreted by *Pachymedusa dacnicolor* by mass spectrometry. (TIF)

S6 Fig Far-UV CD spectra of DMS-DA6-OH and DMS-DA6-NH_2_ in DPC and SDS micelles, and DMPC/DMPG vesicles. (TIF)

S7 Fig NMR chemical shift deviations (CSD) of Cα resonances for DMS-DA6 bound to SDS micelles. (TIF)

S8 Fig Summary of sequential and medium-range HN/HN and HN/Hα NOEs for DMS-DA6 bound to SDS micelles. (TIF)

S1 Table NMR and refinement statistics for the structure calculation of DMS-DA6 bound to SDS micelles. (DOCX)

S2 Table Sequence alignment of DMS-DA6 with the closest sequences of plasticins. The percentages of similarity were obtained with ClustalW2 on http://aps.unmc.edu/AP/main.php.
(DOCX)

S1 Protocol Detailed Materials and methods for the isolation, purification, and sequencing of DMS-DA6 from the skin exudate of *Pachymedusa dacnicolor*. (DOCX)

## References

[1] Ghosh C, Haldar J. Membrane-active small molecules: designs inspired by antimicrobial peptides. ChemMedChem. 2015;10(10):1606–24. 10.1002/cmdc.201500299 . 26386345

[2] Wang G, Li X, Wang Z. APD2: the updated antimicrobial peptide database and its application in peptide design. Nucleic Acids Res. 2009;37(Database issue):D933–7. 10.1093/nar/gkn823 ; PubMed Central PMCID: PMCPMC2686604. 18957441 PMC2686604

[3] Conlon JM, Iwamuro S, King JD. Dermal cytolytic peptides and the system of innate immunity in anurans. Ann N Y Acad Sci. 2009;1163:75–82. 10.1111/j.1749-6632.2008.03618.x . 19456329

[4] Ponti D, Mignogna G, Mangoni ML, De Biase D, Simmaco M, Barra D. Expression and activity of cyclic and linear analogues of esculentin-1, an anti-microbial peptide from amphibian skin. Eur J Biochem. 1999;263(3):921–7. 10469159 10.1046/j.1432-1327.1999.00597.x

[5] Rinaldi AC. Antimicrobial peptides from amphibian skin: an expanding scenario: Commentary. Curr Opin Chem Biol. 2002;6(6):799–804. . 12470734 10.1016/s1367-5931(02)00401-5

[6] Epand RM, Epand RF. Bacterial membrane lipids in the action of antimicrobial agents. J Pept Sci. 2011;17(5):298–305. 10.1002/psc.1319 . 21480436

[7] Malanovic N, Lohner K. Gram-positive bacterial cell envelopes: The impact on the activity of antimicrobial peptide. Biochim Biophys Acta. 2016;1858(5):936–46. 10.1016/j.bbamem.2015.11.004 . 26577273

[8] Dennison SR, Phoenix DA. Influence of C-terminal amidation on the efficacy of modelin-5. Biochemistry. 2011;50(9):1514–23. 10.1021/bi101687t . 21241054

[9] Kim JY, Park SC, Yoon MY, Hahm KS, Park Y. C-terminal amidation of PMAP-23: translocation to the inner membrane of Gram-negative bacteria. Amino Acids. 2010 Epub 2010/06/01. 10.1007/s00726-010-0632-1 . 20512598

[10] Nguyen LT, Chau JK, Perry NA, de Boer L, Zaat SAJ, Vogel HJ. Serum stabilities of short tryptophan- and arginine-rich antimicrobial peptide analogs. PLoS One. 2010;5(9). Medline:. 20844765 10.1371/journal.pone.0012684 PMC2937036

[11] Meneses EP, Villa-Hernandez O, Hernandez-Orihuela L, Castro-Franco R, Pando V, Aguilar MB, et al Peptidomic analysis of the skin secretions of the frog Pachymedusa dacnicolor. Amino Acids. 2010;40(1):113–22. Epub 2010/03/31. 10.1007/s00726-010-0564-9 . 20352461

[12] André S, Washington SK, Darby E, Vega M, Filip AD, Ash NS, et al Structure−Activity Relationship-based Optimization of Small Temporin-SHf Analogs with Potent Antibacterial Activity. ACSchemicalBiology. 2015;10:2257–66. 10.1021/acschembio.5b00495 26181487

[13] Cerovsky V, Budesinsky M, Hovorka O, Cvacka J, Voburka Z, Slaninova J, et al Lasioglossins: three novel antimicrobial peptides from the venom of the eusocial bee Lasioglossum laticeps (Hymenoptera: Halictidae). Chembiochem. 2009;10(12):2089–99. 10.1002/cbic.200900133 . 19591185

[14] Niklison-Chirou MV, Dupuy F, Saavedra L, Hebert E, Banchio C, Minahk C, et al Microcin J25-Ga induces apoptosis in mammalian cells by inhibiting mitochondrial RNA-polymerase. Peptides. 2011;32(4):832–4. 10.1016/j.peptides.2011.01.003 . 21256173

[15] da Silva AV, De Souza BM, Dos Santos Cabrera MP, Dias NB, Gomes PC, Neto JR, et al The effects of the C-terminal amidation of mastoparans on their biological actions and interactions with membrane-mimetic systems. Biochim Biophys Acta. 2014;1838(10):2357–68. 10.1016/j.bbamem.2014.06.012 . 24955498

[16] Mura M, Wang J, Zhou Y, Pinna M, Zvelindovsky AV, Dennison SR, et al The effect of amidation on the behaviour of antimicrobial peptides. Eur Biophys J. 2016;45(3):195–207. 10.1007/s00249-015-1094-x ; PubMed Central PMCID: PMCPMC4796345. 26745958 PMC4796345

[17] Auvynet C, Joanne P, Bourdais J, Nicolas P, Lacombe C, Rosenstein Y. Dermaseptin DA4, although closely related to dermaseptin B2, presents chemotactic and Gram-negative selective bactericidal activities. Febs J. 2009;276(22):6773–86. 10.1111/j.1742-4658.2009.07392.x . 19843179

[18] Riordan JF, Vallee BL. [41] acetylation. Methods Enzymol. 1972;25:494–9. 10.1016/S0076-6879(72)25045-5 . 23014430

[19] Karas M, Hillenkamp F. Laser desorption ionization of proteins with molecular masses exceeding 10,000 daltons. Anal Chem. 1988;60(20):2299–301. . 3239801 10.1021/ac00171a028

[20] Bugg TD, Wright GD, Dutka-Malen S, Arthur M, Courvalin P, Walsh CT. Molecular basis for vancomycin resistance in Enterococcus faecium BM4147: biosynthesis of a depsipeptide peptidoglycan precursor by vancomycin resistance proteins VanH and VanA. Biochemistry. 1991;30(43):10408–15. . 1931965 10.1021/bi00107a007

[21] Auvynet C, El Amri C, Lacombe C, Bruston F, Bourdais J, Nicolas P, et al Structural requirements for antimicrobial versus chemoattractant activities for dermaseptin S9. Febs J. 2008;275(16):4134–51. Epub 2008/07/19. EJB6554 [pii] 10.1111/j.1742-4658.2008.06554.x . 18637027

[22] Sani MA, Henriques ST, Weber D, Separovic F. Bacteria may cope differnetly to similar membrane damage caused by the Australian tree frog antimicrobial peptide maculatin1.1. The Journal of Biochemical Chemistry. 2015;290(32):19853–62. 10.1074/jbc.M115.643262 PMC4528145 26100634

[23] Joanne P, Falord M, Chesneau O, Lacombe C, Castano S, Desbat B, et al Comparative study of two plasticins: specificity, interfacial behavior, and bactericidal activity. Biochemistry. 2009;48(40):9372–83. 10.1021/bi901222p . 19711984

[24] Carlier L, Joanne P, Khemtemourian L, Lacombe C, Nicolas P, El Amri C, et al Investigating the role of GXXXG motifs in helical folding and self-association of plasticins, Gly/Leu-rich antimicrobial peptides. Biophys Chem. 2015;196:40–52. 10.1016/j.bpc.2014.09.004 . 25291467

[25] Hawrani A, Howe RA, Walsh TR, Dempsey CE. Thermodynamics of RTA3 peptide binding to membranes and consequences for antimicrobial activity. Biochim Biophys Acta. 2010;1798(6):1254–62. 10.1016/j.bbamem.2010.03.017 ; PubMed Central PMCID: PMCPMC2877818. 20346912 PMC2877818

[26] Shen Y, Bax A. Protein backbone and sidechain torsion angles predicted from NMR chemical shifts using artificial neural networks. J Biomol NMR. 2013;56(3):227–41. 10.1007/s10858-013-9741-y ; PubMed Central PMCID: PMCPMC3701756. 23728592 PMC3701756

[27] Guntert P, Mumenthaler C, Wuthrich K. Torsion angle dynamics for NMR structure calculation with the new program DYANA. J Mol Biol. 1997;273(1):283–98. 10.1006/jmbi.1997.1284 . 9367762

[28] Rinaldi AC, Di Giulio A, Liberi M, Gualtieri G, Oratore A, Bozzi A, et al Effects of temporins on molecular dynamics and membrane permeabilization in lipid vesicles. J Pept Res. 2001;58(3):213–20. . 11576327 10.1034/j.1399-3011.2001.00896.x

[29] Dennison SR, Mura M, Harris F, Morton LH, Zvelindovsky A, Phoenix DA. The role of C-terminal amidation in the membrane interactions of the anionic antimicrobial peptide, maximin H5. Biochim Biophys Acta. 2015;1848(5):1111–8. 10.1016/j.bbamem.2015.01.014 . 25640709

[30] Chen HC, Brown JH, Morell JL, Huang CM. Synthetic magainin analogues with improved antimicrobial activity. FEBS Lett. 1988;236(2):462–6. . 3410055 10.1016/0014-5793(88)80077-2

[31] Marien E, Meister M, Muley T, Fieuws S, Bordel S, Derua R, et al Non-small cell lung cancer is characterized by dramatic changes in phospholipid profiles. Int J Cancer. 2015;137(7):1539–48. 10.1002/ijc.29517 ; PubMed Central PMCID: PMCPMC4503522. 25784292 PMC4503522

[32] Huang L, Chen D, Wang L, Lin C, Ma C, Xi X, et al Dermaseptin-PH: A Novel Peptide with Antimicrobial and Anticancer Activities from the Skin Secretion of the South American Orange-Legged Leaf Frog, Pithecopus (Phyllomedusa) hypochondrialis. Molecules. 2017;22(10). 10.3390/molecules22101805 . 29064402 PMC6151546

[33] Shi D, Hou X, Wang L, Gao Y, Wu D, Xi X, et al Two Novel Dermaseptin-Like Antimicrobial Peptides with Anticancer Activities from the Skin Secretion of Pachymedusa dacnicolor. Toxins (Basel). 2016;8(5). 10.3390/toxins8050144 . 27187467 PMC4885059

[34] Dennison SR, Harris F, Bhatt T, Singh J, Phoenix DA. The effect of C-terminal amidation on the efficacy and selectivity of antimicrobial and anticancer peptides. Mol Cell Biochem. 2009;332(1–2):43–50. 10.1007/s11010-009-0172-8 . 19513817

[35] Saravanan R, Li X, Lim K, Mohanram H, Peng L, Mishra B, et al Design of short membrane selective antimicrobial peptides containing tryptophan and arginine residues for improved activity, salt-resistance and biocompatibility. Biotechnol Bioeng. 2013 10.1002/bit.25003 . 23860860

[36] Deslouches B, Phadke SM, Lazarevic V, Cascio M, Islam K, Montelaro RC, et al De novo generation of cationic antimicrobial peptides: influence of length and tryptophan substitution on antimicrobial activity. Antimicrob Agents Chemother. 2005;49(1):316–22. 10.1128/AAC.49.1.316-322.2005 ; PubMed Central PMCID: PMCPMC538858. 15616311 PMC538858

[37] Li J, Koh JJ, Liu S, Lakshminarayanan R, Verma CS, Beuerman RW. Membrane Active Antimicrobial Peptides: Translating Mechanistic Insights to Design. Front Neurosci. 2017;11:73 10.3389/fnins.2017.00073 ; PubMed Central PMCID: PMCPMC5306396. 28261050 PMC5306396

[38] Sun Y, Dong W, Sun L, Ma L, Shang D. Insights into the membrane interaction mechanism and antibacterial properties of chensinin-1b. Biomaterials. 2015;37:299–311. 10.1016/j.biomaterials.2014.10.041 . 25453959

[39] Ladokhin AS, Jayasinghe S, White SH. How to measure and analyze tryptophan fluorescence in membranes properly, and why bother? Anal Biochem. 2000;285(2):235–45. 10.1006/abio.2000.4773 . 11017708

[40] Walrant A, Correia I, Jiao CY, Lequin O, Bent EH, Goasdoue N, et al Different membrane behaviour and cellular uptake of three basic arginine-rich peptides. Biochim Biophys Acta. 2011;1808(1):382–93. 10.1016/j.bbamem.2010.09.009 . 20920465

[41] Malanovic N, Leber R, Schmuck M, Kriechbaum M, Cordfunke RA, Drijfhout JW, et al Phospholipid-driven differences determine the action of the synthetic antimicrobial peptide OP-145 on Gram-positive bacterial and mammalian membrane model systems. Biochimica et biophysica acta. 2015;1848(10 Pt A):2437–47. Medline:. 26210299 10.1016/j.bbamem.2015.07.010

[42] Conlon JM. Structural diversity and species distribution of host-defense peptides in frog skin secretions. Cell Mol Life Sci. 2011;68(13):2303–15. 10.1007/s00018-011-0720-8 . 21560068 PMC11114843

[43] Nicolas P, Amiche M. The dermaseptins In: Kastin AJ, editor. Handbook of biologically active peptides. San Diego, CA: Elsevier; 2006 p. 295–304.

[44] El Amri C, Lacombe C, Zimmerman K, Ladram A, Amiche M, Nicolas P, et al The plasticins: membrane adsorption, lipid disorders, and biological activity. Biochemistry. 2006;45(48):14285–97. 10.1021/bi060999o . 17128968

[45] Roelants K, Fry BG, Ye L, Stijlemans B, Brys L, Kok P, et al Origin and functional diversification of an amphibian defense Peptide arsenal. PLoS Genet. 2013;9(8):e1003662 10.1371/journal.pgen.1003662 . 23935531 PMC3731216

[46] Bechinger B, Zasloff M, Opella SJ. Structure and orientation of the antibiotic peptide magainin in membranes by solid-state nuclear magnetic resonance spectroscopy. Protein Sci. 1993;2(12):2077–84. 10.1002/pro.5560021208 ; PubMed Central PMCID: PMCPMC2142334. 8298457 PMC2142334

[47] Nguyen LT, Haney EF, Vogel HJ. The expanding scope of antimicrobial peptide structures and their modes of action. Trends Biotechnol. 2011;29(9):464–72. 10.1016/j.tibtech.2011.05.001 . 21680034

[48] Brannan AM, Whelan WA, Cole E, Booth V. Differential scanning calorimetry of whole Escherichia coli treated with the antimicrobial peptide MSI-78 indicate a multi-hit mechanism with ribosomes as a novel target. PeerJ. 2015;3:e1516 10.7717/peerj.1516 ; PubMed Central PMCID: PMCPMC4690349. 26713257 PMC4690349

[49] Wimley WC. Describing the mechanism of antimicrobial peptide action with the interfacial activity model. ACS Chem Biol. 2010;5(10):905–17. 10.1021/cb1001558 ; PubMed Central PMCID: PMCPMC2955829. 20698568 PMC2955829

[50] Neelay OP, Peterson CA, Snavely ME, Brown TC, TecleMariam AF, Campbell JA, et al Antimicrobial peptides interact with peptidoglycan. Journal of Molecular Structure. 2017;1146:329–36.

