# Supplementary figures and images for: Peptidoglycan potentiates the membrane disrupting effect of the carboxyamidated form of DMS-DA6, a Gram-positive selective antimicrobial peptide isolated from Pachymedusa dacnicolor skin
Source: PLoS One. 2018 Oct 16;13(10):e0205727. doi: 10.1371/journal.pone.0205727 (PMC6191125; doi:10.1371/journal.pone.0205727)

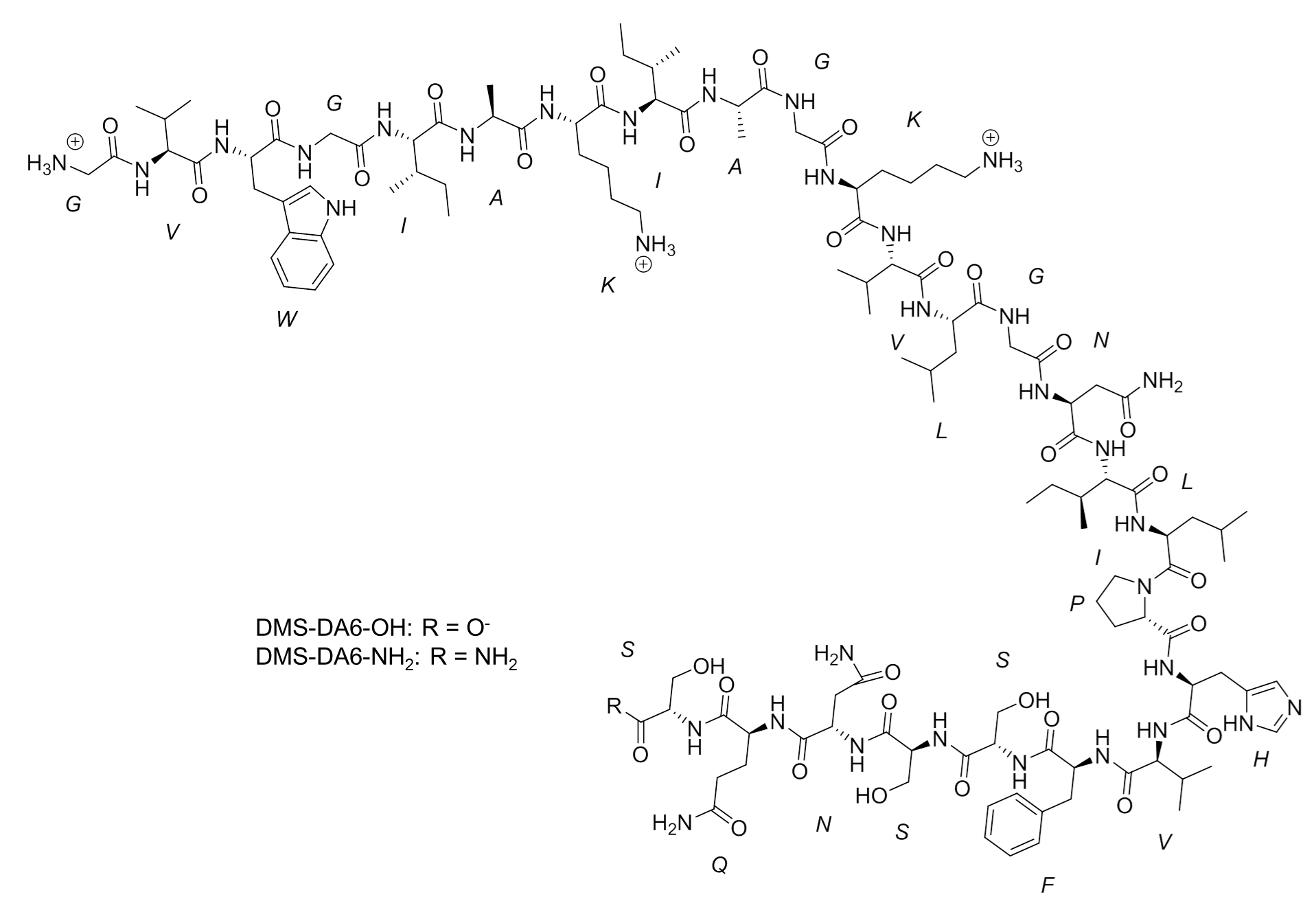

Supplement: S1 Fig — (TIF) [file pone.0205727.s001.tif]

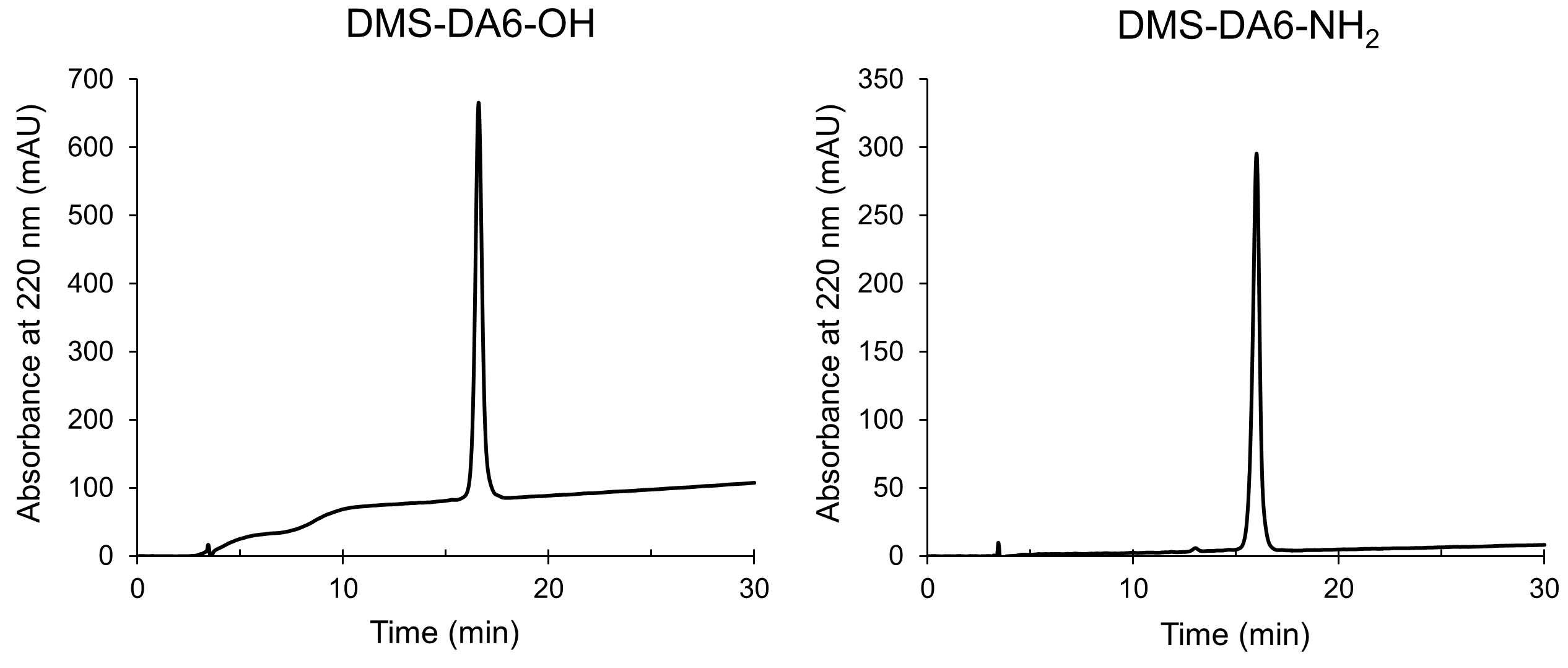

Supplement: S2 Fig — (TIF) [file pone.0205727.s002.tif]

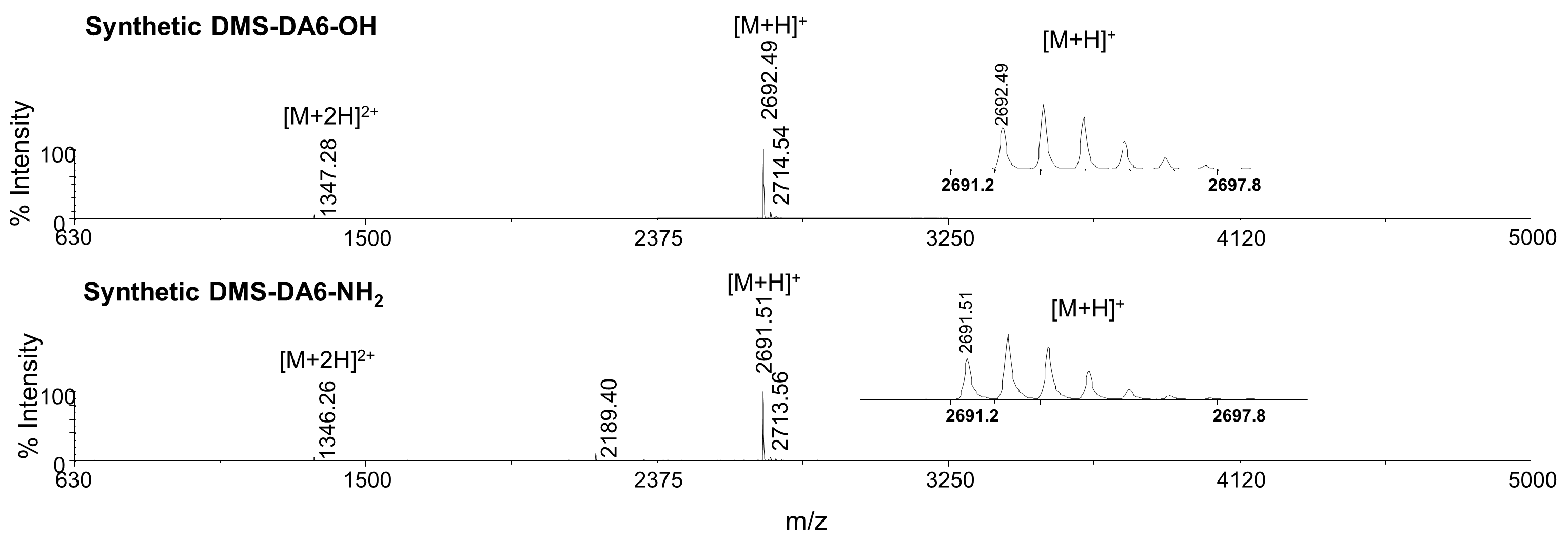

Supplement: S3 Fig — (TIF) [file pone.0205727.s003.tif]

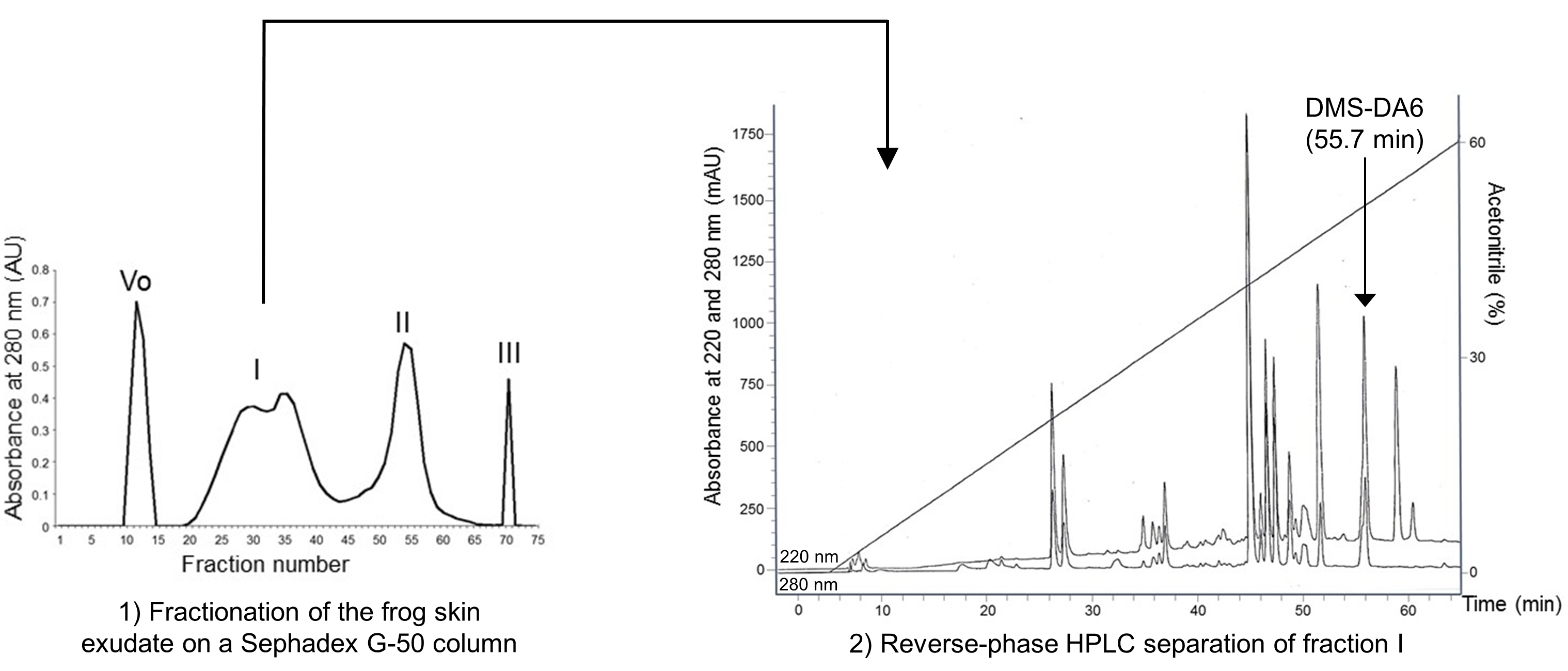

Supplement: S4 Fig — Fractionation profile of the skin exudate on a Sephadex G-50 column followed by reverse-phase HPLC separation using a semi-preparative column. (TIF) [file pone.0205727.s004.tif]

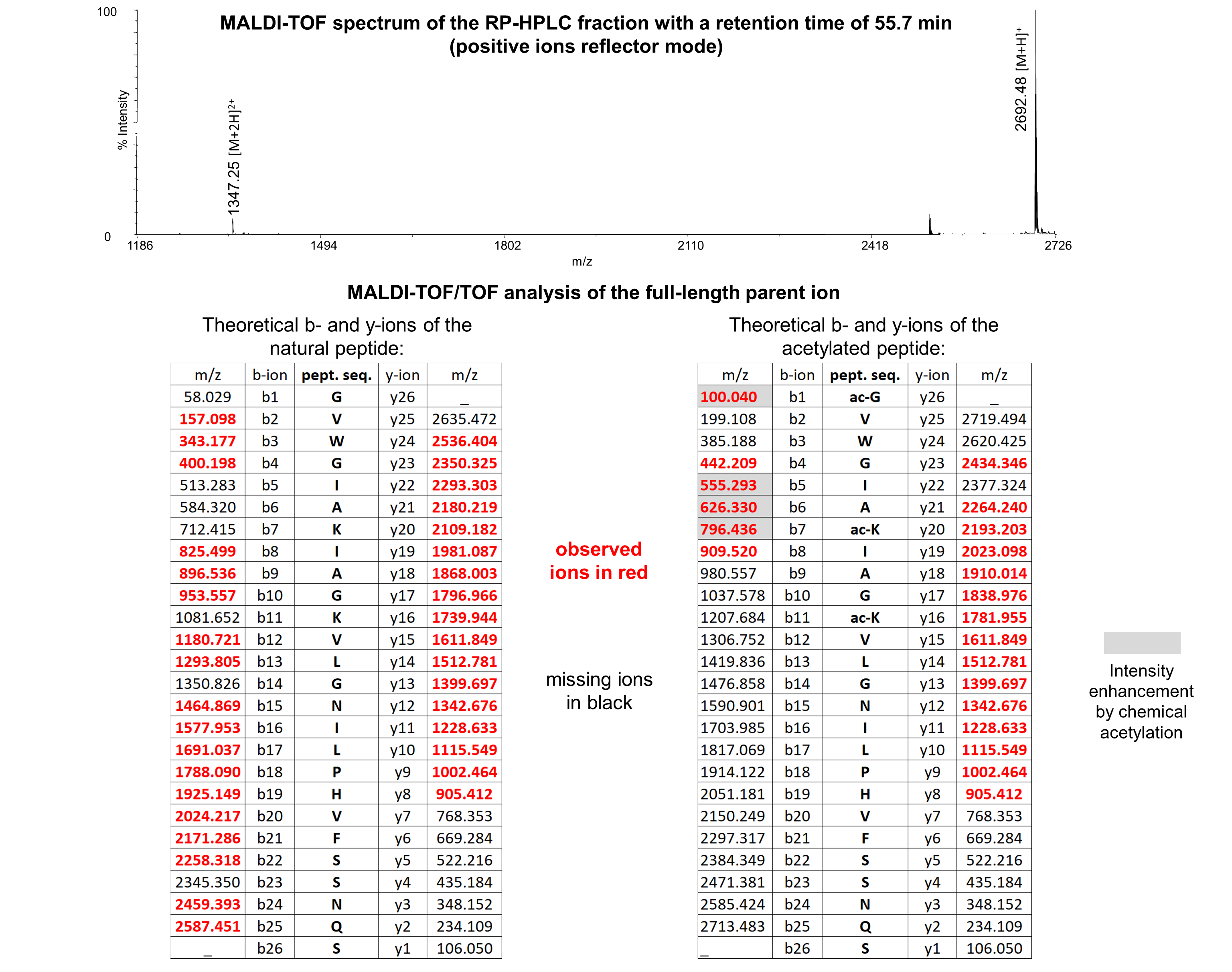

Supplement: S5 Fig — (TIF) [file pone.0205727.s005.tif]

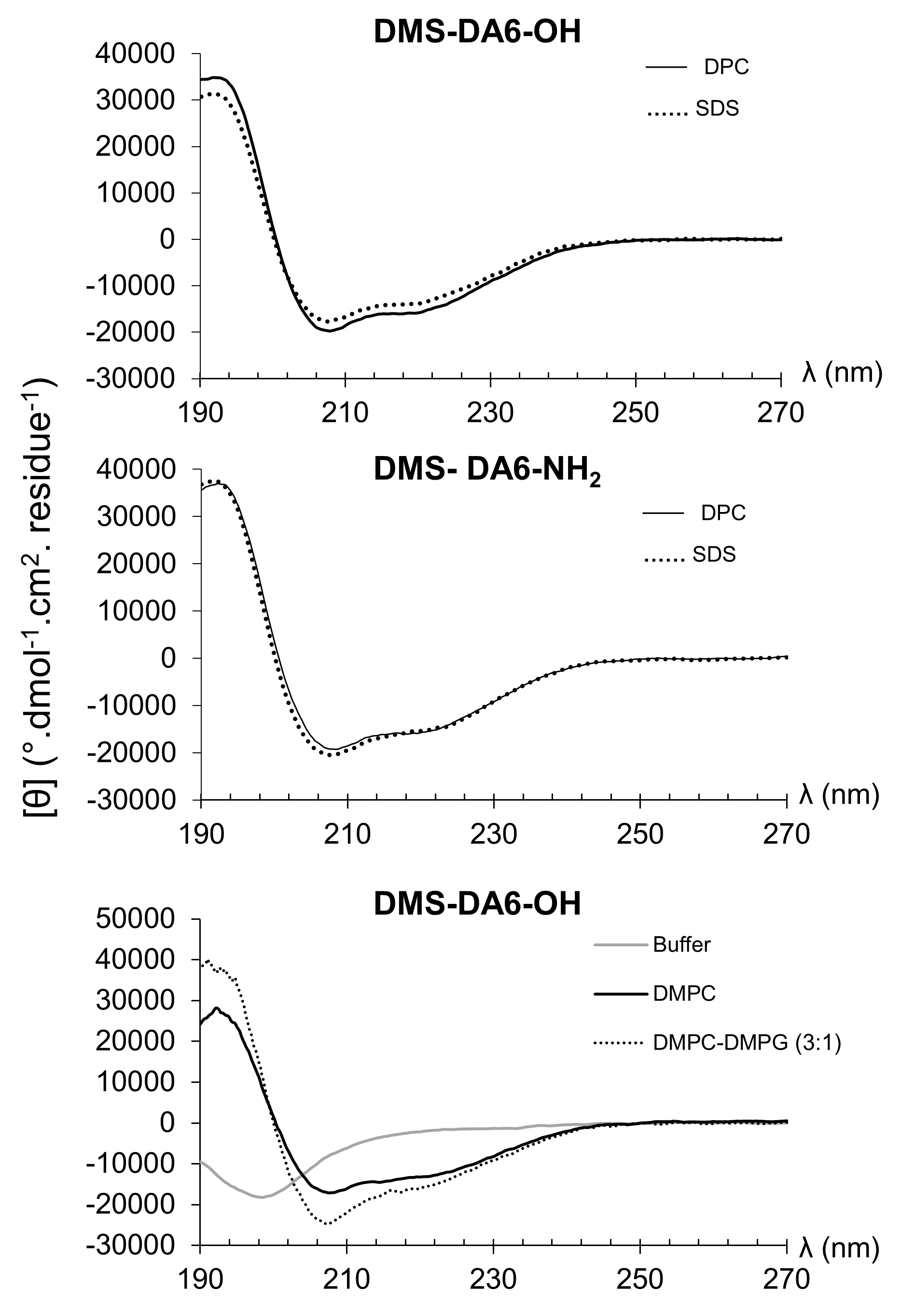

Supplement: S6 Fig — (TIF) [file pone.0205727.s006.tif]

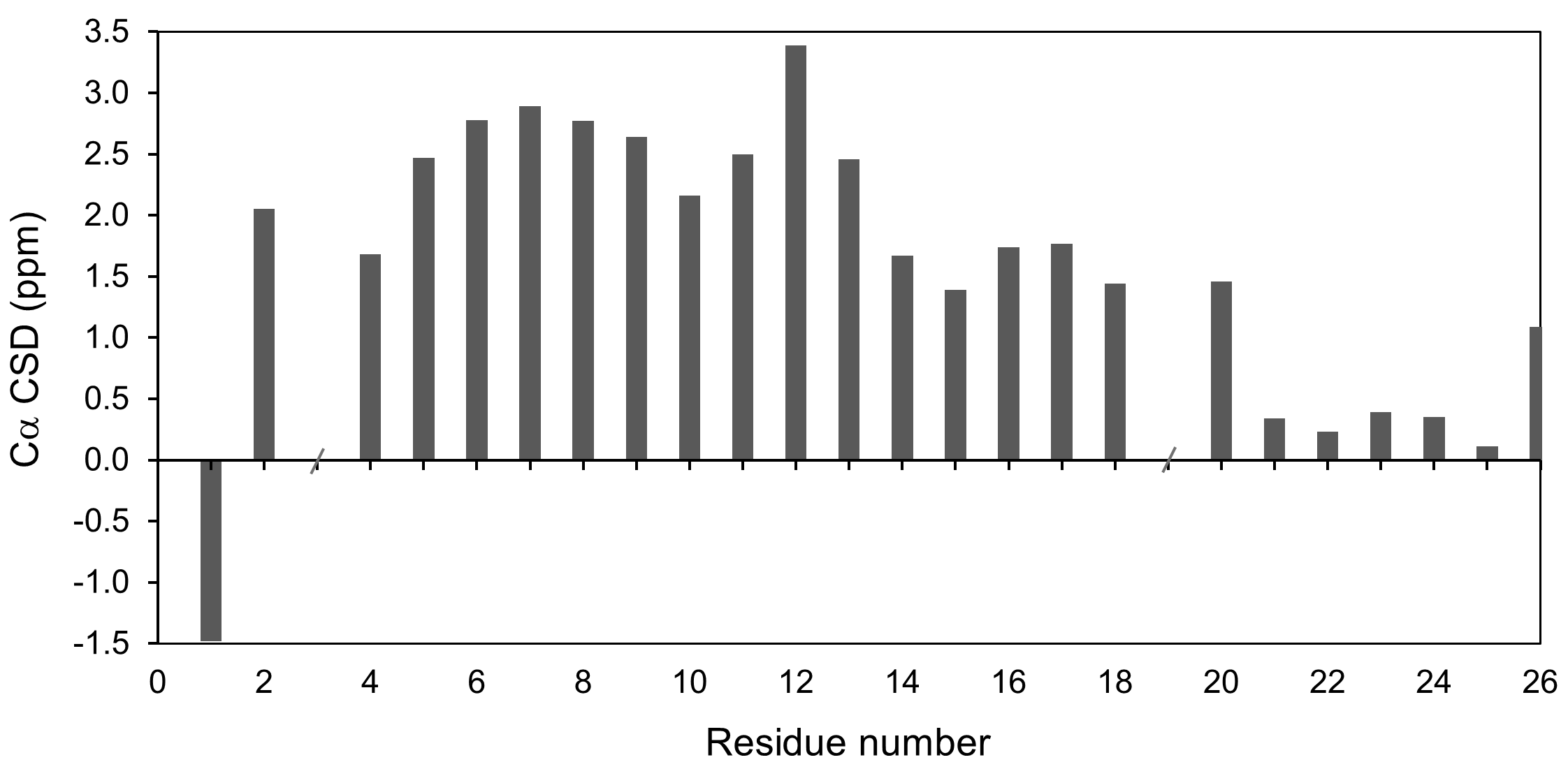

Supplement: S7 Fig — (TIF) [file pone.0205727.s007.tif]

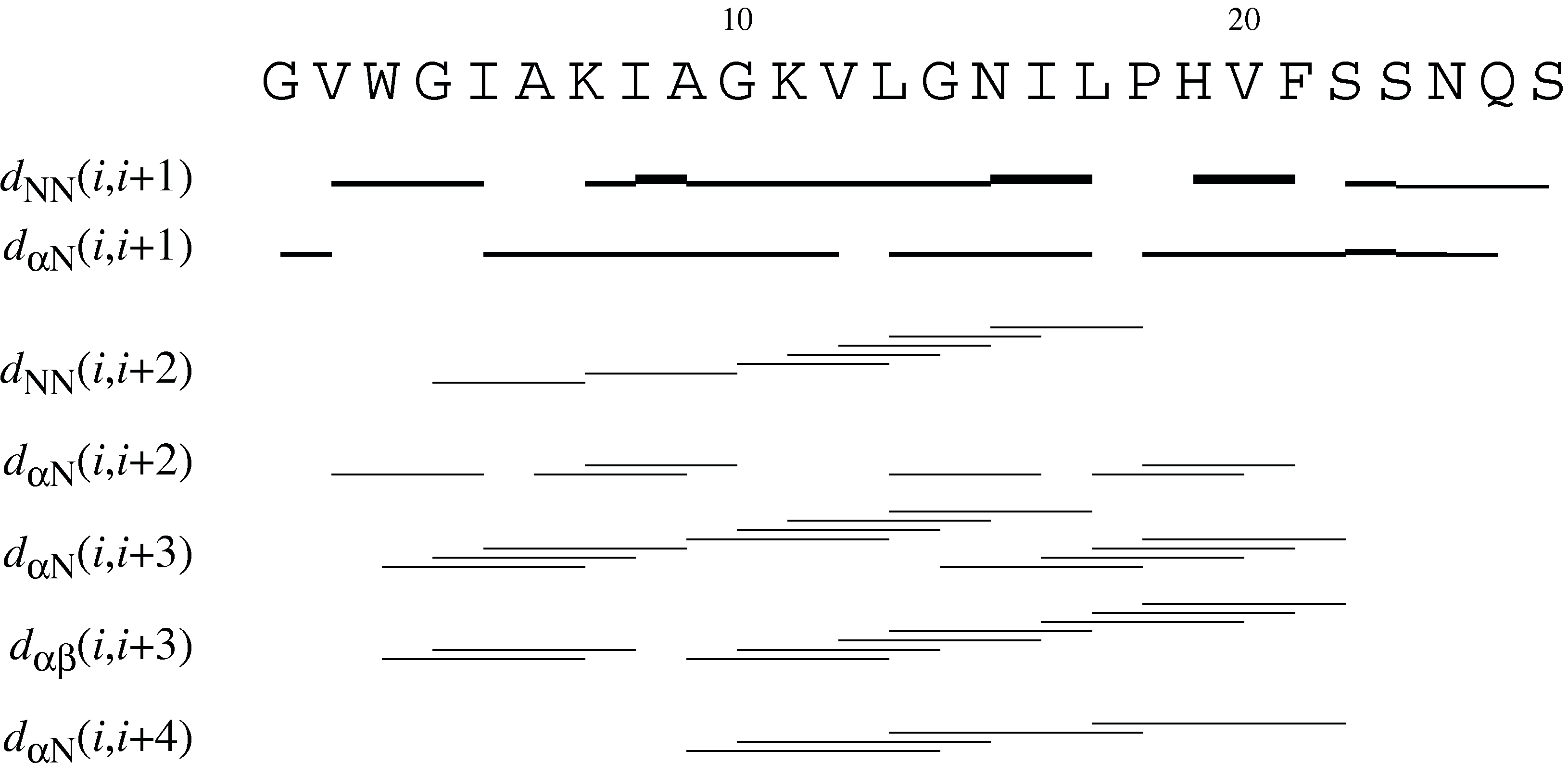

Supplement: S8 Fig — (TIF) [file pone.0205727.s008.tif]
