# Supplementary material for: Peptidoglycan potentiates the membrane disrupting effect of the carboxyamidated form of DMS-DA6, a Gram-positive selective antimicrobial peptide isolated from Pachymedusa dacnicolor skin
Source: PLoS One. 2018 Oct 16;13(10):e0205727. doi: 10.1371/journal.pone.0205727 (PMC6191125; doi:10.1371/journal.pone.0205727)
Supplement: S1 Table — (DOCX) [file pone.0205727.s009.docx]

**Experimental restraints**

Number of nOe-derived distance restraints 516

Ambiguous 41

Unambiguous 475

Intra-residue and sequential 375

Medium-range (2 ≤ |*i – j*| ≤ 5) 100

Long-range (|*i – j*| > 5) 0

Number of dihedral angle restraints 36

φ 18

ψ 18

**Structural statistics**

Violations

No. distance restraints

> 0.5 Å 0 ± 0

> 0.3 Å 0.4 ± 0.5

> 0.1 Å 11.0 ± 2.1

No. dihedral angle restraints (> 5°) 0 ± 0

RMS deviations from ideal geometry

Bond (Å) 0.004

Angle (°) 0.51

Improper (°) 1.60

RMS deviations on restraints

Distance (Å) 0.03

Dihedral (°) 0.47

Structural dispersion (residues 3-21)

Backbone (Å) 0.4

Heavy atoms (Å) 0.8

Ramachandran plot (residues 3-21)

Most favored 87.2%

Additionally allowed 12.8%

Generously allowed 0 %

Disallowed regions 0 %

MolProbity all-atom Clashscore 14.88
