# Supplementary material for: Peptidoglycan potentiates the membrane disrupting effect of the carboxyamidated form of DMS-DA6, a Gram-positive selective antimicrobial peptide isolated from Pachymedusa dacnicolor skin
Source: PLoS One. 2018 Oct 16;13(10):e0205727. doi: 10.1371/journal.pone.0205727 (PMC6191125; doi:10.1371/journal.pone.0205727)
Supplement: S2 Table — The percentages of similarity were obtained with ClustalW2 on http://aps.unmc.edu/AP/main.php. (DOCX) [file pone.0205727.s010.docx]

| **Peptide** | **Sequence** | **Similarity percentage** | **Reference** |
| --- | --- | --- | --- |
| **DMS-DA6** | **GVWG-IAKIAGKVLGNI-L-PHVFSSNQS** |  | [11] |
| **Plasticin PD36K** | **GVVTDLLKTAGKLLGN--L—-VGS-LSG** | 46.42 | [44] |
| **Plasticin PD36KF** | **GVVTDLLKTAGKLLGN--L—-FGS-LSG** | 46.42 | [44] |
| **CPF-St5** | **GVFGLLAKAALK--GASKLIPHLLPSRQQ** | 44.82 | [45] |
| **Plasticin B1** | **GLVTSLIKGAGKLLG-G-L-FGSVTGGQS** | 44.82 | [44] |
