# Supplementary material for: Peptidoglycan potentiates the membrane disrupting effect of the carboxyamidated form of DMS-DA6, a Gram-positive selective antimicrobial peptide isolated from Pachymedusa dacnicolor skin
Source: PLoS One. 2018 Oct 16;13(10):e0205727. doi: 10.1371/journal.pone.0205727 (PMC6191125; doi:10.1371/journal.pone.0205727)
Supplement: S1 Protocol — (DOCX) [file pone.0205727.s011.docx]

Fresh skin exudate (200 µL) of the frog *Pachymedusa dacnicolor* was recovered by gently squeezing the later-dorsal portion of a frog skin, resuspended in deionized water and centrifuged (400 x g, 15 min). The supernatant was fractionated by size exclusion chromatography on a Sephadex G-50 fine column (60 x 0.75 cm) eluted with 10% acetic acid. Absorbance was monitored at 280 nm. Three main fractions (I, II, and III) were obtained and fraction I was further fractionated by reverse-phase HPLC on a semi-preparative column (Nucleosil 5 µm C18, 250 x 10 mm) using a solvent system composed of water containing 0.1% trifluoroacetic acid as solvent A and, acetonitrile containing 0.07% trifluoroacetic acid as solvent B. The column was eluted at 5 mL.min^-1^ with a 0-60% linear gradient of solvent B for 65 min. RP-HPLC fractions were then submitted to an antimicrobial activity assay; the fraction with a retention time of 55.7 min showed antimicrobial activity against the Gram-positive bacteria *S. aureus* ATCC 6538, but not against the Gram-negative bacteria *E. coli* ATCC 35218. This RP-HPLC fraction was further analyzed by mass spectrometry for peptide identification and *de novo* sequencing.

The MALDI-TOF analysis of the RP-HPLC fraction revealed a peptide with a m/z ratio in the 2.0 to 3.5 kDa mass range, consistent with a Dermaseptin-like peptide. Identification of singly and doubly protonated ions at m/z 2692.48 and m/z 1347.25, respectively, suggested the presence of at least one residue with a high proton affinity in the gas phase. Because of the relatively low mass accuracy of the MALDI-TOF/TOF technique (> 20 ppm), acetylation was performed to differentiate Lys from Gln residues (the latter do not react) before tandem MS fragmentation of the full-length parent ion, since these residues have near isobaric masses. The MALDI-TOF/TOF analysis of the acetylated peptide revealed the presence of two lysines in the peptide sequence. Acetylation also helped *de-novo* sequencing (higher sequence coverage) by substantially increasing the production and the intensity of the lower m/z b-ions and enhancing in source-decay (ISD) fragmentation. By combining the MS/MS information collected from the acetylated and non-acetylated parent peptides, we obtained an almost complete b-ions series leading to a 26-residue peptide sequence (GVWGXAKXAGKVXGNXXPHVFSSNQS) containing 5 ambiguities between isobaric Leu and Ile residues (denoted as X). The assignment of Leu and Ile residues was deduced by sequence comparison with one of the Dermaseptin-like peptides from *Pachymedusa dacnicolor*, previously reported by Meneses *et al*. (2010, Amino Acids, 40:113-122) by combining full Edman sequencing on a tryptic digest and MS fragmentation using CID and High energy Collision Dissociation (HCD).
